# Worldwide research trends on the *Helicobacter pylori*–gut microbiome nexus: a bibliometric analysis

**DOI:** 10.3389/fimmu.2026.1794021

**Published:** 2026-04-16

**Authors:** Liwei Li, Fuqing Cai, Shikai Liu, Peng Peng, Jingrong Liang, Zheng Liu, Hengyuan Xu, Weijiu Mo, Jiamin Qin, Sufan Tang, Huaqiang Ruan, Jinxiu Zhang, Chenghai Liang, Shiquan Liu, Mengbin Qin, Rongbin Qin, Feilong Luo, Guang Xiong, Chongze Yang, Yan Geng, Jun Zou, Jiean Huang

**Affiliations:** 1 Department of Gastroenterology, The Second Affiliated Hospital of Guangxi Medical University, Nanning, China; 2 Department of Gastroenterology, 923 Hospital of People's Liberation Army Joint Logistics Support Force, Nanning, China; 3 Department of Geriatric Endocrinology and Metabolism, The First Affiliated Hospital of Guangxi Medical University, Nanning, China; 4 Department of Radiology, Guangxi Hospital Division of The First Affiliated Hospital, Sun Yat-sen University, Nanning, China

**Keywords:** bibliometric, gut microbiome, *Helicobacter pylori*, immunotherapy, tumor microbiome

## Abstract

**Introduction:**

The impact of *Helicobacter pylori* (*H. pylori*) on the gastrointestinal tract ecosystem has been widely investigated beyond the stomach. Researchers have made considerable progress in understanding the relationship between *H. pylori* infection, gut microbiome dysbiosis, and systemic effects in recent years. This study aimed to explore the prospects and developing trends in the field of the *H. pylori*–gut microbiome nexus from a bibliometric perspective.

**Methods:**

Articles were collected from the Web of Science Core Collection, Scopus, and PubMed (2000–2025) and analyzed using bibliometrix, VOSviewer, and CiteSpace. Analysis of 1,592 publications reveals a distinct three-phase evolutionary structure in the field.

**Results and discussion:**

Geographically led by China and the USA, the research focus has undergone a paradigm shift: evolving from an initial “infection and eradication” phase, through a transitional “dysbiosis and ecological intervention” phase, to the current “tumor–immunity axis” hotspot. Recent high-strength citation bursts for terms like “*Fusobacterium nucleatum*” and “immunity” underscore this transformation, indicating that academic attention has moved beyond local gastric pathogen control to understanding *H. pylori*’s systemic role in modulating tumor microenvironments and therapeutic responses. This bibliometric analysis maps the field’s rapid growth trajectory, highlighting its value for guiding future precision oncology and microecological strategies.

## Graphical Abstract

**Figure:**
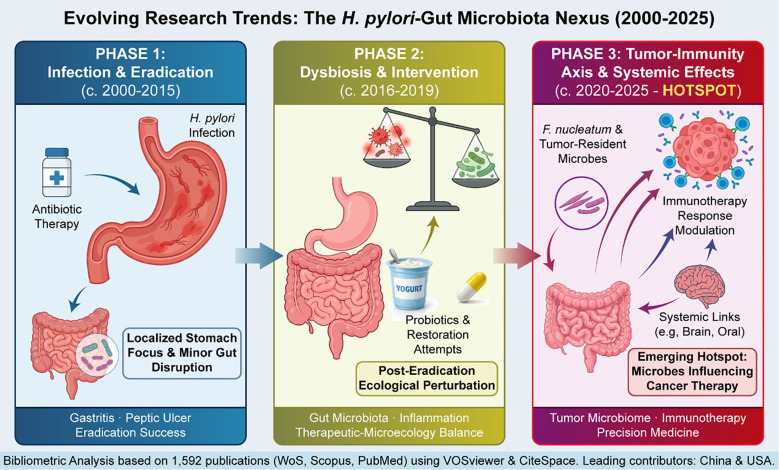

## Highlights

- This study presents a comprehensive bibliometric analysis of research on the *Helicobacter pylori*–gut microbiota nexus from 2000 to 2025.
- Global research output has increased rapidly since 2020, with China and the United States leading in productivity while exhibiting distinct collaboration patterns.
- Research hotspots have shifted from infection and eradication toward the tumor–microbiome–immunity axis, indicating emerging links to cancer and immunotherapy.

## Introduction

*Helicobacter pylori* (*H. pylori*), a gram-negative microaerophilic bacterium, chronically colonizes the stomach and remains one of the most widespread human pathogens (1–3). Since its discovery in 1982, it has been established as a cause of chronic gastritis, peptic ulceration and gastric cancer, earning classification as a Group 1 carcinogen (4, 5). Survival under acidic conditions is achieved through several tolerance systems, making it a useful model for host–pathogen research (6, 7).

With the rise of microbiome science, it is now clear that *H. pylori* is not restricted to the stomach but alters microbial communities throughout the gastrointestinal tract (8–10). It competes for nutrients (11) and binding sites (12), secretes antimicrobial products (13, 14) and induces inflammation that reshapes local environments (15–17). Yet evidence remains contradictory: some studies report major microbiome shifts (18–20), while others find little effect (21), reflecting differences in design and methodology.

With the expansion of microbiome research, increasing attention has been directed toward the broader consequences of *Helicobacter pylori* infection beyond the stomach. *H. pylori* colonizes exclusively the gastric mucosa, yet its impact extends indirectly to the intestinal microbiome through several complex mechanisms, including infection-induced alterations in gastric acid secretion, inflammatory signaling, and host physiological responses, which together may reshape downstream microbial environments (22, 23). Importantly, the effects of *H. pylori* infection on gastric acid secretion are heterogeneous across patients and disease patterns (24). Corpus-predominant gastritis is commonly associated with hypochlorhydria, potentially permitting enhanced survival and transmission of acid-sensitive microorganisms (24, 25), whereas antrum-predominant gastritis may be accompanied by increased acid output, imposing selective pressure on intestinal microbial communities (26). Such divergent physiological contexts may help explain the inconsistent microbiome alterations reported across studies.

Beyond acid-mediated effects, *H. pylori*–induced gastric inflammation may exert systemic immunological consequences, including altered cytokine signaling and regulatory T-cell responses, which could indirectly influence distal microbial communities (27). Emerging evidence further suggests that gut microbiome composition can modulate host immune tone and disease susceptibility, extending to cancer-related immune responsiveness (28). These findings raise ongoing debate regarding whether *H. pylori*–associated dysbiosis exerts context-dependent protective or deleterious effects, underscoring a shift from stomach-centered pathogenicity toward a systems-level host–microbiome interaction framework (29).

Modern sequencing and multiomics approaches (30, 31), which include 16S rRNA profiling (32, 33), shotgun metagenomics (34, 35), metatranscriptomics (36) and metabolomics (37), allow far more detailed mapping of microbial ecology and its interaction with *H. pylori*. These insights may refine diagnosis (38), highlight biomarkers of treatment response, and inspire alternatives to antibiotics (39), including phage therapy (40–42), though ecological consequences are not yet well defined.

This study undertakes a bibliometric survey of research on *H. pylori* and the gut microbiome, tracking thematic development, methodological trends and collaboration networks, with the aim of elucidating knowledge gaps and guiding future directions. Ultimately, this comprehensive mapping is essential for bridging the gap between ecological insights and clinical practice, paving the way for precision eradication strategies that preserve the gut ecosystem.

## Materials and methods

### Data collection and search strategy

For this bibliometric study, we searched Web of Science Core Collection, Scopus, and PubMed to capture publications addressing associations between *Helicobacter pylori* and the gut microbial ecosystem from January 1, 2000 to December 10, 2025. Web of Science Core Collection was used as the primary dataset for bibliometric analyses, whereas Scopus and PubMed were queried in parallel to examine database coverage and to verify that the overall evidence base was comparable across indexing platforms. The Web of Science search was implemented in the Topic field using TS=(“Helicobacter pylori” OR “H. pylori”) AND TS=((“gut” NEAR/3 microb*) OR “gut microbiome” OR “gut microbiota” OR “intestinal microbiota” OR “intestinal microbiome” OR “fecal microbiota”); the Scopus search was run in TITLE-ABS-KEY fields using (TITLE-ABS-KEY(“Helicobacter pylori” OR “H. pylori”)) AND (TITLE-ABS-KEY((“gut” W/3 microb*) OR “gut microbiome” OR “gut microbiota” OR “intestinal microbiota” OR “intestinal microbiome” OR “fecal microbiota”)); and the PubMed strategy combined MeSH with title and abstract terms as ((“Helicobacter pylori”[MeSH] OR “Helicobacter pylori”[tiab] OR “H. pylori”[tiab]) AND (“gut microbiome”[tiab] OR “gut microbiota”[tiab] OR “intestinal microbiota”[tiab] OR “intestinal microbiome”[tiab] OR “fecal microbiota”[tiab] OR (“gut”[tiab] AND (microbiome[tiab] OR microbiota[tiab] OR microb*[tiab])))). The search strategy was designed to balance specificity and coverage within the H. pylori–gut microbiome research domain. Pathogen-specific terms (“Helicobacter pylori” OR “H. pylori”) were combined with intestinal microbiome–related descriptors, including “gut microbiome,” “gut microbiota,” “intestinal microbiota,” “intestinal microbiome,” and “fecal microbiota.” In Web of Science and Scopus, proximity operators (NEAR/3 or W/3) were applied to link “gut” with microbiome-related terms (microb*), capturing variant expressions while maintaining contextual relevance. In PubMed, MeSH terms were integrated with title and abstract keywords using equivalent microbiome-related expressions. This keyword framework was refined through pilot searches to ensure consistent retrieval of microbiome-focused studies across databases.

**Figure 1:**
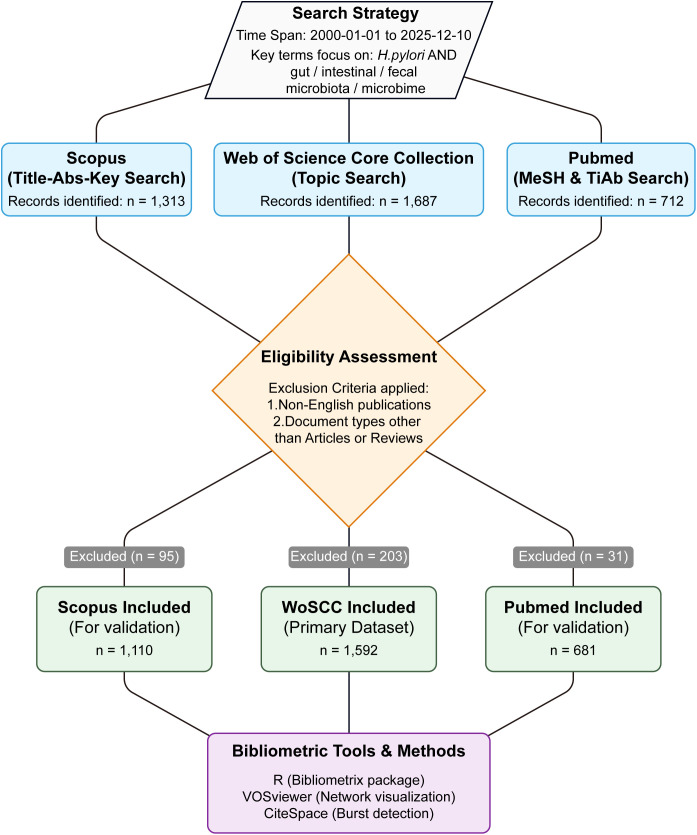
Flowchart of the literature search and screening process. This figure illustrates the workflow used for literature retrieval and study selection in the bibliometric analysis. Records were obtained from three major databases: Web of Science Core Collection, Scopus, and PubMed, covering publications from January 1, 2000 to December 10, 2025. After the initial retrieval (Web of Science: n = 1,687; Scopus: n = 1,313; PubMed: n = 712), duplicate removal and eligibility screening were performed. Only English-language articles and reviews focusing on the relationship between *Helicobacter pylori* infection and the gut microbiome were included. Publications focusing exclusively on treatment regimens or clinical outcomes without microbiome-related endpoints were excluded. After screening, 1,592 records from Web of Science were retained for the primary bibliometric analysis, while 1,110 records from Scopus and 681 records from PubMed were used for cross-database validation.

Only English-language articles and reviews were considered. The searches initially returned 1,687 records in Web of Science, 1,313 in Scopus, and 712 in PubMed; after applying the eligibility criteria and screening, 1,592, 1,110, and 681 records, respectively, were retained for analysis (**Figure 1**). Eligible records were restricted to English-language articles and reviews explicitly addressing the relationship between *H. pylori* infection and gut microbial composition, diversity, or function. Publications focusing solely on eradication regimens, drug efficacy, or clinical outcomes without microbiome-related endpoints were excluded. The complete screening workflow, including record counts at each stage, is summarized in **Figure 1**.

Journal Impact Factors and quartile rankings were taken from Clarivate’s Journal Citation Reports (JCR 2024).

This study did not involve human participants or animals, and therefore ethical approval and informed consent were not required. As this is a bibliometric analysis, prospective registration was not required.

This study was conducted in compliance with the TITAN Guidelines 2025 for declaration and use of AI in scholarly publishing (43).

### Bibliometric analysis

Bibliometric profiling followed a multi-tool workflow that combined quantitative description, network visualization and thematic evolution. Collaboration and conceptual structures were mapped with VOSviewer v1.6.20, which generated co-authorship and keyword co-occurrence networks using fractional counting and minimum thresholds of three documents per author or institution and five co-occurrences per keyword. To identify influential references and nascent research fronts, CiteSpace v6.3.R1 performed reference- and keyword-citation-burst analysis across yearly time slices from 2000 to 2025. Data analysis and visualization were completed in R (v4.5.0) with the bibliometrix package supplemented by readxl, dplyr, scales, tidyr, stringr, country code, and ggplot2. The complete R scripts and parameter details are available from the corresponding author upon reasonable request. The author impact metrics presented in this study, including the H-index, were calculated based exclusively on the retrieved dataset (Local H-index) rather than the authors’ overall publication history (Global H-index). This approach ensures that the metrics reflect the authors’ specific contributions and influence within the analyzed research domain.

### Statistical analysis

Because variables were skewed, we used nonparametric procedures. Two-group comparisons employed the two-sided Wilcoxon rank-sum test, while three-or-more-group comparisons used the Kruskal–Wallis test followed by two-sided pairwise Wilcoxon *post hoc* tests. P values from multiple pairwise tests were adjusted using the Benjamini–Hochberg (BH) method. Continuous data are summarized as median (IQR), and α = 0.05 (two-sided). For one-sample proportion checks, exact binomial tests with BH adjustment across countries were used.

## Results

### Study retrieval and screening

In total, the database searches yielded 1,687 records in Web of Science, 1,313 in Scopus, and 712 in PubMed (**Figure 1**). After restricting to English-language articles and reviews, 1,592 WoS records were included in the bibliometric analyses, while 1,110 Scopus records and 681 PubMed records were retained for cross-database comparison (**Figure 1**).

### Global publication trends

**Figure 2:**
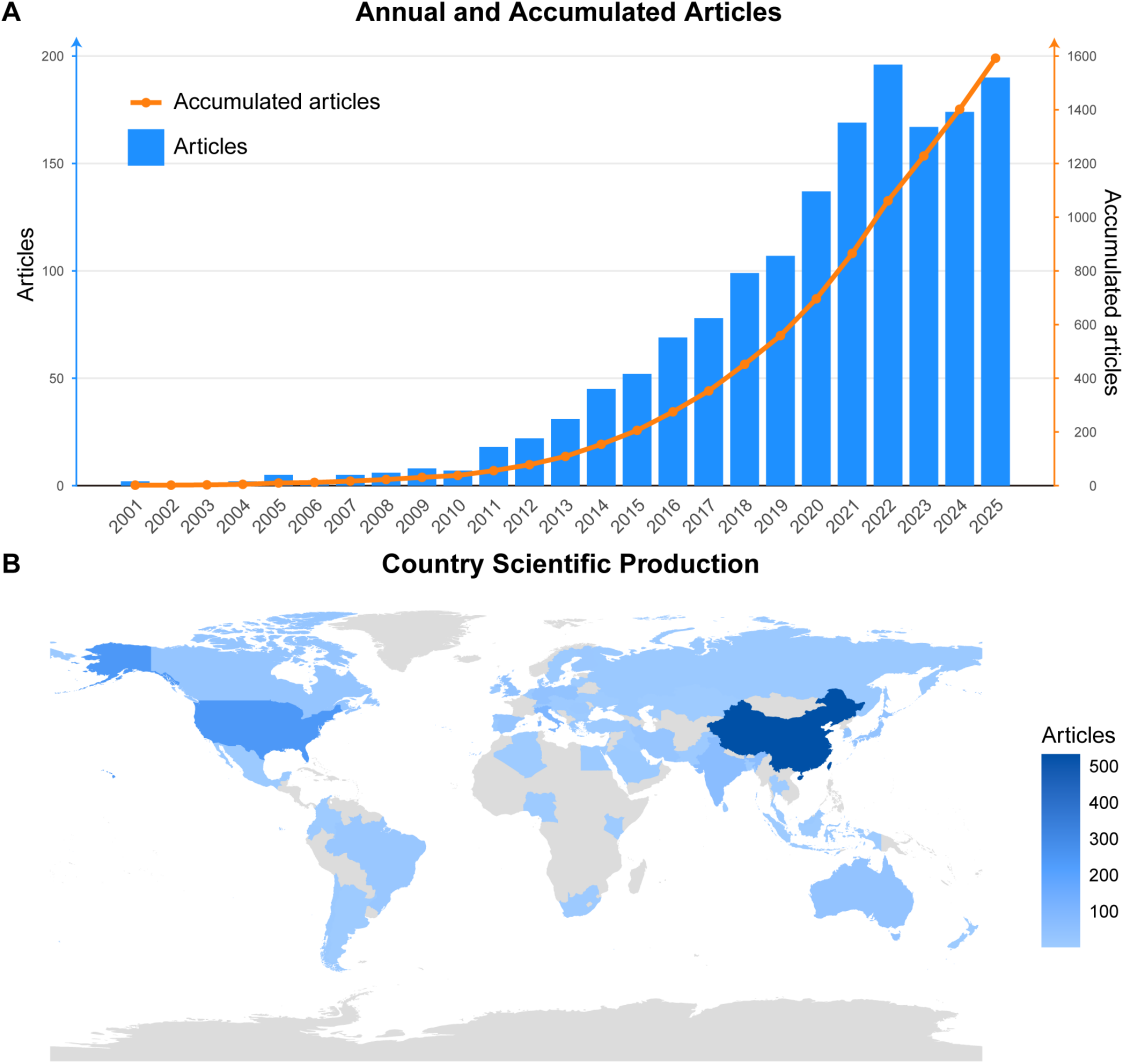
Global publication trends and geographical distribution of research on the *H. pylori*–gut microbiota nexus. **(A)** Annual publication output and cumulative growth of publications indexed in the Web of Science Core Collection from 2000 to 2025. The figure demonstrates a progressive increase in scientific output over time, with a particularly rapid expansion after 2015 and a peak publication volume around 2022. The cumulative curve highlights the accelerating growth of the research field in recent years. **(B)** Global distribution of scientific production by country or region. The world map visualizes the geographic distribution of publications, with color intensity representing the number of articles contributed by each country. Countries with darker shading indicate higher research productivity, highlighting major contributors such as China and the United States.

The WoS dataset contains 1,592 publications which demonstrate a steady increase in yearly publication numbers from start to finish of the research period. Although the search period commenced in 2000, no publications were retrieved for that year. Between 2000 and 2009, only 31 papers were published. In the following period, the output grew to 123 papers, which researchers published between 2010 and 2014. Growth became much more pronounced after 2015, specifically, 405 papers were published in 2015–2019. The most substantial expansion occurred in 2020–2025, when a total of 1,033 papers were published, accounting for 64.9% of the WoS corpus. The research output achieved its peak during 2022 when scientists published 196 papers and maintained this high level through subsequent years with 167, 179 and 190 publications in 2023, 2024 and 2025 respectively (**Figure 2**). Scopus (n = 1,110) and PubMed (n = 681) showed the same overall temporal pattern, and were used to support the WoS-based assessment of global publication trends (**Supplementary Figures 1**, **2**).

### Article types and study characteristics

**Figure 3:**
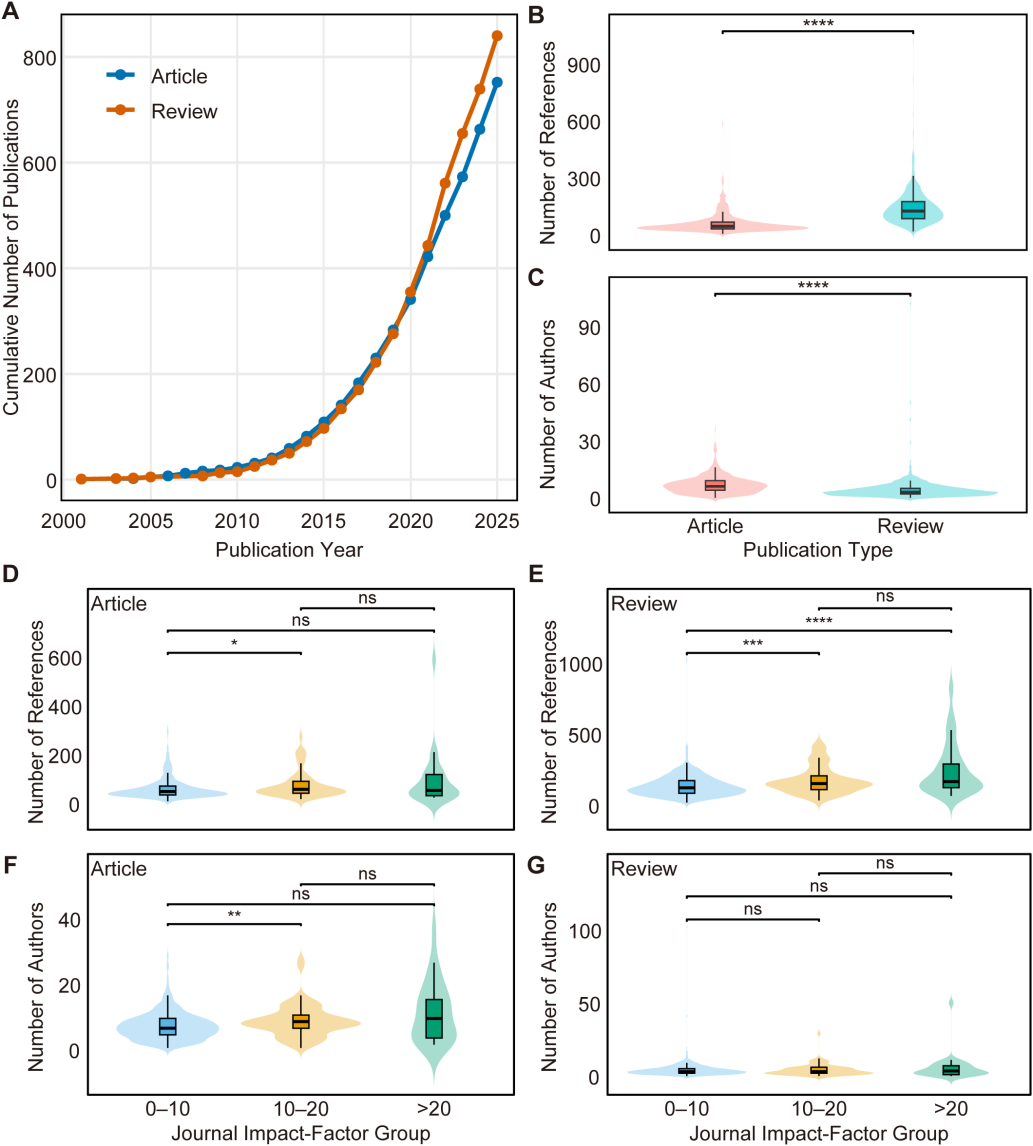
Article types and study characteristics. **(A)** Cumulative publication trends comparing research articles and review articles within the dataset. **(B, C)** Comparative analysis of reference counts and numbers of authors between document types. Boxplots display the distribution of references cited and the size of author teams, demonstrating that research articles generally involve larger author teams, whereas review articles typically contain significantly more references. **(D, E)** Distribution of reference counts stratified by journal impact factor **(IF)** categories (IF 0–10, IF 10–20, IF >20) for research articles **(D)** and review articles **(E)**, illustrating differences in citation density across journal tiers. **(F, G)** Distribution of author team sizes across journal impact factor groups for research articles **(F)** and review articles **(G)**, showing variations in collaboration scale associated with journal prestige. The asterisks indicate the following levels of statistical significance from pairwise Wilcoxon tests with Benjamini–Hochberg correction: * p < 0.05; ** p < 0.01; *** p < 0.001; **** p < 0.0001; ns = not significant.

A total of 1,592 publications from the WoS Core Collection were included, comprising 752 research articles and 840 reviews (**Figure 3A**). Overall, research articles were produced by significantly larger teams than reviews, whereas reviews cited substantially more references (both p < 0.001; **Figures 3B, C**). These patterns were independently reproduced in the Scopus dataset (n = 1,110), confirming their robustness across databases (**Supplementary Figure 3B**).

When stratified by journal impact factor (IF), reference counts in research articles showed a limited association with journal tier. Only articles published in mid-range journals (IF 10–20) exhibited a modest but significant increase in references compared with the baseline (IF 0–10), whereas no further expansion was observed in the highest impact group (IF >20; **Figure 3D**). In contrast, review articles demonstrated a clear saturation pattern, with reference counts increasing from low- to mid-impact journals but plateauing between the two upper IF tiers (**Figure 3E**).

Authorship patterns displayed database-specific behavior at the upper impact range. In the WoS dataset, research articles showed a significant expansion in team size only in the IF 10–20 group, while articles published in IF >20 journals did not differ significantly from the low-impact baseline (**Figure 3F**). Review articles exhibited minimal variation in authorship across all IF tiers (**Figure 3G**). By comparison, Scopus data revealed a sustained increase in research article team size with rising journal impact, reaching the largest teams in the IF >20 group (**Supplementary Figure 3C**). Conversely, Scopus review articles showed a contraction in team size at the highest impact tier (**Supplementary Figure 3D**).

### Geography and international collaboration

**Figure 4:**
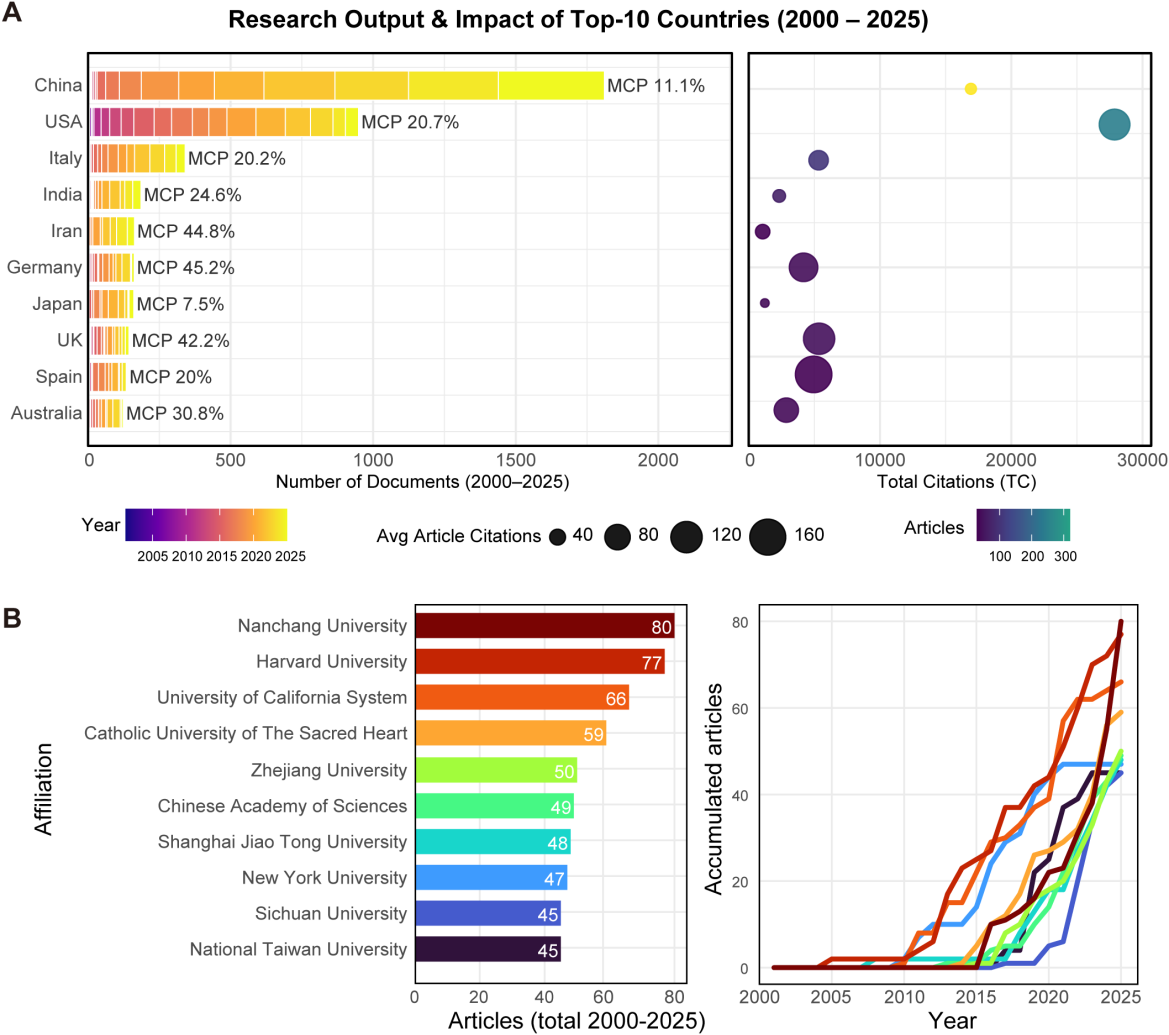
Research output and impact of top countries and institutions. **(A)** Comparative analysis of the top 10 most productive countries in the *H. pylori*–gut microbiome research field. Bars represent the total number of publications, while additional indicators illustrate the level of international collaboration (multiple-country publications, MCP) and citation impact metrics. **(B)** Publication output and temporal accumulation curves for the top 10 contributing institutions worldwide. The graph displays both total publication counts and the trajectory of research productivity over time, revealing the rise of major academic centers and shifts in institutional leadership within the field.

In terms of publication volume (**Figure 4A**), China was the most productive country in the WoS corpus, accounting for one-third of the total output (33.4%, n=532). It was followed by the USA (15.2%), Italy (6.5%), and India (4.3%). These top four nations together contributed 59.4% of the global total. However, high publication volume did not correlate with high international collaboration. China’s Multiple Country Publications (MCP) ratio was only 11.1%, indicating a reliance on domestic research—a pattern also seen in Japan (7.5%). In comparison, European countries like the UK and Germany exhibited much stronger collaboration networks, with MCP ratios of 42.2% and 45.2%, respectively.

Institutionally (**Figure 4B**), the landscape highlights the rise of Chinese research hubs. Nanchang University took the top spot with 80 publications, just ahead of Harvard University (n=77) and the University of California System (n=66). And Italian institutions also performed well. The accumulation curves illustrate a key trend: while Harvard maintained steady productivity, Nanchang University’s output surged in the last decade, allowing it to overtake established western institutions. These findings were similar to Scopus (**Supplementary Figure 4**) and PubMed (**Supplementary Figure 5**) datasets, confirming China’s leading output and Nanchang University’s high productivity.

### Journal distribution, impact factors and JCR quartiles

**Figure 5:**
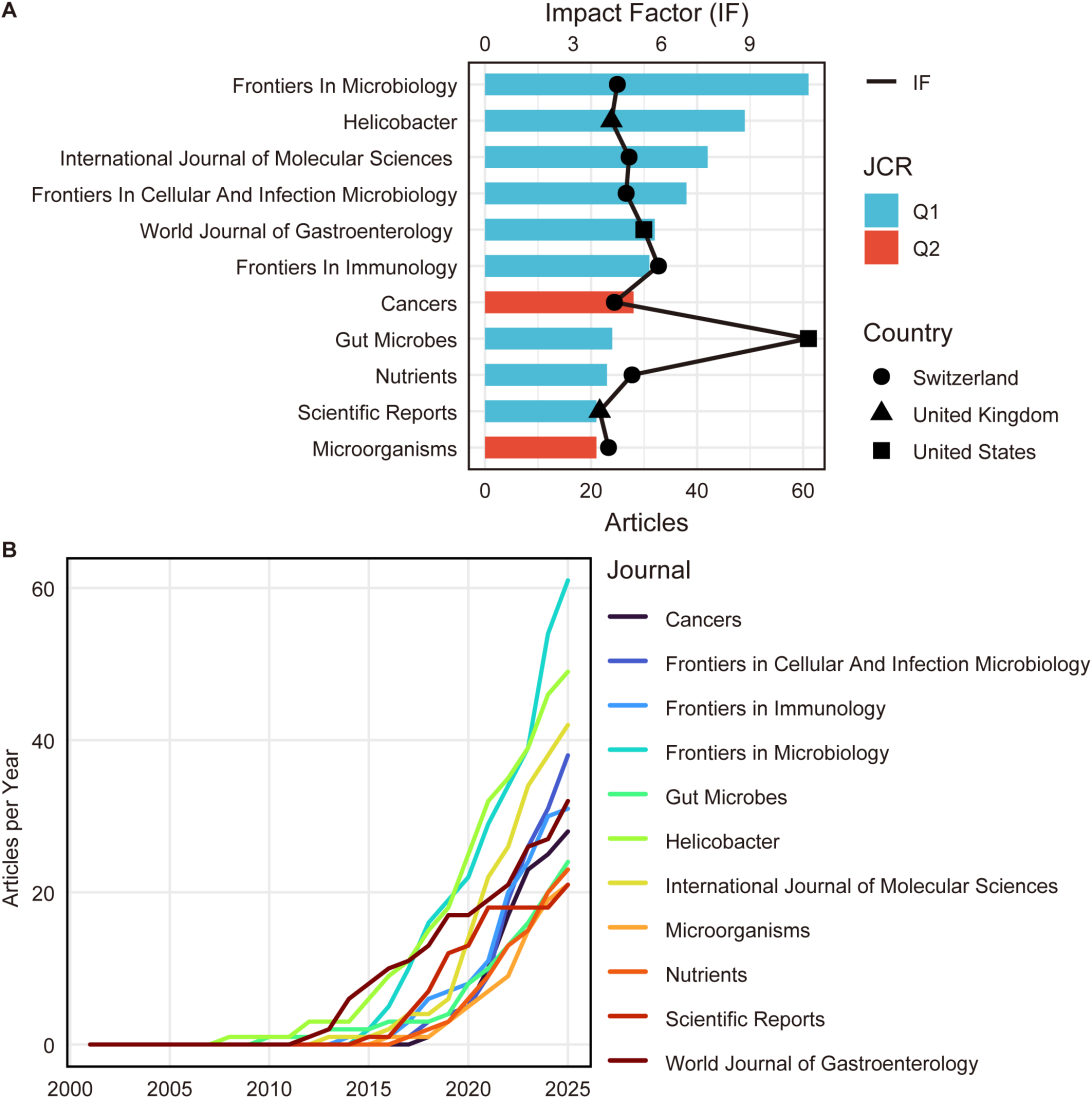
Journal distribution and publication dynamics. **(A)** The top 11 journals publishing studies related to the *H. pylori*–gut microbiota nexus. Bar charts represent the total number of publications contributed by each journal, with colors indicating Journal Citation Reports (JCR) quartile rankings. The line plot indicates the Impact Factor of each journal. Different shapes represent the geographic location of the journal publisher. **(B)** Temporal publication trends of the top 11 journals, illustrating how the contribution of leading journals to this research field has evolved over time.

The top 10 publishing venues were largely Q1-ranked journals. In WoS, *Frontiers in Microbiology* (n=61, IF 4.5) and *Helicobacter* (n=49, IF 4.3) were the most productive, while *Gut Microbes* exhibited the highest Impact Factor (IF 11.0) (**Figure 5**). Although Scopus (**Supplementary Figure 6**) and PubMed (**Supplementary Figure 7**) rankings slightly favored Helicobacter, cross-database analysis confirmed a highly consistent core journal landscape.

### Author collaboration networks

Analysis of the WoS dataset (**Supplementary Figure 8A**) highlights a centralized yet fragmented collaboration landscape. The most prominent feature is a dense collaborative network (red cluster) revolving around He, Cong, Zhu, Yin, and Lu, Nonghua. While the blue cluster (e.g., Hu, Yi) maintains bridging ties to this core, other subgroups—specifically the green (e.g., Lai, Yongkang) and yellow (e.g., Zeng, Liping) clusters—operate as largely independent entities with weak outward linkages. Validation via Scopus (**Supplementary Figure 8B**) and PubMed (**Supplementary Figure 8C**) confirmed this structural pattern, demonstrating high consistency in identifying both the central collaborative ‘triad’ and the detached peripheral groups.

### Author productivity, citation impact, and temporal activity

**Figure 6:**
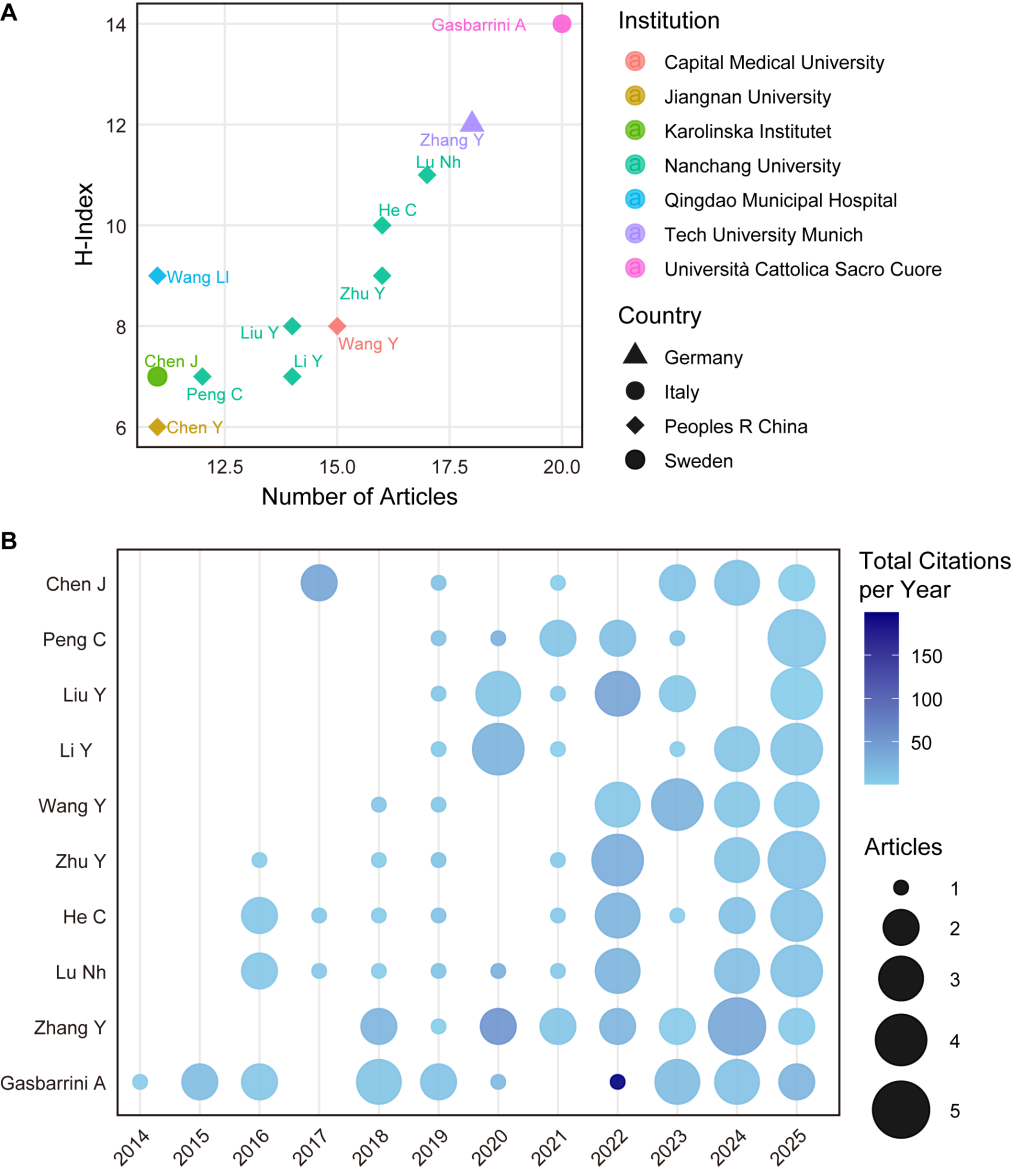
Scientific production and impact of top authors. **(A)** Relationship between the number of publications and the local H-index among the most productive authors in the dataset. Each point represents an individual author, illustrating the association between research productivity and citation impact within this specific research domain. **(B)** Annual publication output and citation intensity of leading contributors. The temporal distribution of publications highlights the active research periods of major authors and demonstrates how influential researchers have shaped the development of the field over time.

A positive correlation between productivity and local H-index was evident in the top-tier author landscape (**Figure 6A**). Leading contributors such as Gasbarrini A and Zhang Y exemplified this trend, securing dominant positions in both metrics. In the WoS dataset, Gasbarrini A (active since 2014) ranked first with 20 papers and an local H-index of 14, followed closely by Zhang Y (active since 2018) and Lu NH (**Figure 6B**). However, analysis of citation impact reveals a divergence from this quantity-driven pattern. While prolific authors maintained high cumulative scores, they were surpassed in per-article impact by researchers like Blaser MJ (highest total citations: 3,670) and Knight R (highest average: 744.5). This distinction was corroborated by Scopus data (**Supplementary Figure 9**), which mirrored the prominence of Gasbarrini A and Zhang Y but highlighted the ‘quantity-quality’ gap through authors like Wang Y—who, despite a high volume of 34 papers, averaged only 16.0 citations, confirming that maximum output does not invariably guarantee maximal citation impact.

### Highly cited publications

Local citation analysis (**Supplementary Table 1**) revealed two distinct categories of influential records: globally recognized foundational studies and highly specialized core references. The most frequently cited document was the review “The microbiome and cancer” (44), which accumulated 90 local citations and 1,309 global citations. The study by Lertpiriyapong et al. (45) on microbiota-driven gastric carcinogenesis followed with 81 local citations. Notably, high field specificity was observed for the trials by Liou et al. (46) (74 local citations) and Chen et al. (47) (72 local citations); both exhibited Local Citation to Global Citation (LC/GC) ratios exceeding 45%, indicating that nearly half of their total impact originated from this specific research domain. In contrast, the work by Jakobsson et al. (48) on antibiotic effects (70 local citations) demonstrated a predominantly global reach (856 total citations) with a much lower local ratio of 8.18%.

### Reference citation bursts and trend evolution

Citation-burst analysis of 1,592 Web of Science records (2000–2025) identified the top 25 burst references and delineated three phases: metagenomic baselines, *H. pylori*–related dysbiosis, and eradication-linked systemic and long-term effects (**Supplementary Figure 10**).

Phase I (2011–2015) established benchmarks and mechanistic framing: Qin et al. (49) and the Human Microbiome Project (62) defined gut microbial gene and “healthy” reference resources; Schwabe & Jobin (44) summarized inflammation- and genotoxicity-driven carcinogenesis; Eun et al. (51) showed gastric cancer–associated microbial restructuring beyond *H. pylori* dominance, while Arthur et al. (52) and Kostic et al. (53) reinforced inflammation–dysbiosis–tumor parallels from colorectal models.

Phase II (2016–2019) focused on gastric dysbiosis and short-term intervention effects: Aviles-Jimenez et al. (54) and Ferreira et al. (55) characterized cancer-associated shifts (reduced diversity and enrichment of non-*Helicobacter* taxa), with Ferreira et al. (55) proposing a Microbial Dysbiosis Index; Yap et al. (56) documented eradication-associated gut microbiome perturbations, and Oh et al. (57) suggested probiotics may attenuate antibiotic-related changes. Hooi et al. (2) updated global *H. pylori* prevalence, whereas Vétizou et al. (58) and Sivan et al. (59) linked gut commensals to checkpoint immunotherapy efficacy, implying potential extra-gastric consequences of eradication-driven microbiome disruption.

Phase III (2020–2025) extended to long-term outcomes and tumor-resident microbes: Liou et al. (46) reported that post-eradication microbiome and metabolic disturbances are largely transient with longer-term recovery trends; Nejman et al. (60) mapped intratumoural bacteria across cancer types; Sung et al. (61) contextualized the persistent global burden of gastric cancer.

**Figure 7:**
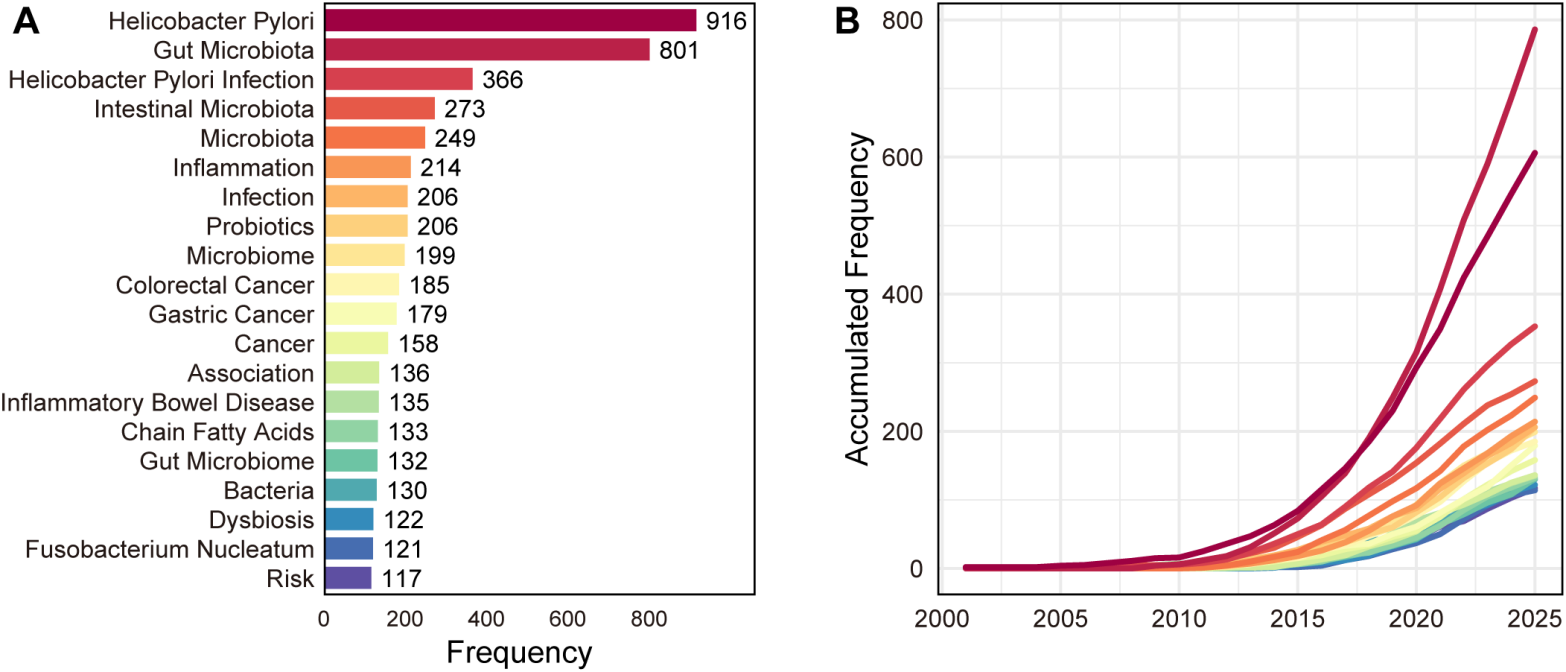
Keyword frequency and temporal trends analysis. **(A)** Distribution of the top 20 high-frequency keywords in the WoS dataset (2000–2025). Core terms are dominated by “*Helicobacter pylori*” (n=916) and “gut microbiota” (n=801), reflecting the field’s foundational orientation. Disease-related endpoints (gastric cancer, colorectal cancer) and mechanistic terms (inflammation, dysbiosis, probiotics) constitute the secondary thematic layer. **(B)** Accumulated growth trends of selected top keywords over time. Keywords showing the most pronounced post-2018 acceleration include “dysbiosis”, “cancer”, and “*Fusobacterium nucleatum*”, indicative of a thematic shift from infection-centered microbiology toward tumor-microbiome and host-immunity research.

Keyword frequency analysis delineates the core thematic structure of *H. pylori*–microbiome research (**Figure 7**, WoS). As expected, “*Helicobacter pylori*” (916) and “gut microbiota” (801) dominate, indicating that the literature is anchored in *H. pylori* infection within an intestinal ecological framework. Infection-related terms remain prominent, including “*Helicobacter pylori* infection” (366) and “infection” (206), while microbiome descriptors such as “intestinal microbiota” (273), “microbiota” (249), and “microbiome” (199) further reinforce the field’s microbiome-centered orientation. In terms of mechanisms and interventions, “inflammation” (214) and “probiotics” (206) are among the most frequent, consistent with sustained interest in host response and microbiota-modulating strategies. Disease endpoints are strongly represented, particularly malignancy-related keywords— “colorectal cancer” (185), “gastric cancer” (179), and “cancer” (158)—together with broader association keywords (e.g., “association” 136 and “risk” 117). Beyond cancer, “inflammatory bowel disease” (135), “dysbiosis” (122), and taxa linked to tumor-associated microbiomes such as *Fusobacterium nucleatum* (121) point to expanding attention to dysbiosis-driven comorbidities and oncogenic microbiota signatures. Scopus validation yielded a highly consistent picture (**Supplementary Figure 11**), with “*Helicobacter pylori*” (934) and “intestine flora” (855) ranking among the top terms alongside “gastrointestinal microbiome” (439) and “Helicobacter infection” (433), and with recurrent emphasis on dysbiosis (372) and probiotic interventions (“probiotic agent” 325). The Scopus keyword set also underscores the predominance of human studies (e.g., “human/humans” 951/646) while retaining substantial nonhuman/animal representation (“nonhuman” 686; “animal/animals” 272/263), supporting the robustness of WoS-derived thematic conclusions. Among these, “dysbiosis”, “*Fusobacterium nucleatum*” and “immunity” showed the most pronounced post-2018 acceleration, suggesting a thematic transition from infection-centered microbiology toward tumor-microbiome and host-immunity research.

The Sankey diagram (**Supplementary Figure 12**) illustrates the alignment between leading institutions, national outputs, and core research themes. Research activity is dominated by Chinese institutions, with additional contributions from major centers in the United States and Europe. The principal thematic flows are centered on Helicobacter pylori and the gut microbiota, with secondary extensions to probiotics, dysbiosis, inflammation, and cancer-related topics. At the country level, flows are concentrated in China and the USA, while broader international participation across Europe and Asia remains evident.

### Keyword co-occurrence network and thematic clusters

The keyword co-occurrence network (**Supplementary Figure 13**) exhibits a highly integrated structure composed of three major thematic clusters with distinct functional orientations. The red cluster centers on Helicobacter pylori and delineates the infection–pathogenesis–management axis, encompassing tightly linked terms related to colonization, eradication, therapy, resistance, and risk assessment. The blue cluster is organized around gut microbiota and represents the intestinal ecological and host-response dimension, integrating disease-related keywords (e.g., inflammatory bowel disease and irritable bowel syndrome), intervention terms (probiotics), and experimental or clinical design signals (e.g., double-blind, *in vitro*). The green cluster captures malignancy-oriented research, dominated by gastric and colorectal cancer, carcinogenesis, immunotherapy, and tumor-associated taxa such as *Fusobacterium nucleatum*. Notably, several high-centrality bridge terms—particularly gut microbiota, inflammation, dysbiosis, and probiotics—link these clusters, indicating mechanistic and translational continuity from gastric infection and immune activation to intestinal ecological disruption and cancer-related outcomes.

### Citation burst analysis of keywords

Citation burst analysis of keywords (**Supplementary Figure 14**) reflects a staged shift in research priorities. The earliest bursts (2001–2015) centered on core gastrointestinal pathogens/diseases and foundational methods, with sustained signals for Escherichia coli, atrophic gastritis, Crohn’s disease, lactic acid bacteria, 16S rRNA, and double-blind trial designs. In the mid-phase (2010–2018), emphasis moved toward microbiome ecology and site-specific context, highlighted by colonization, diversity, community, and stomach, alongside disease-oriented terms such as inflammatory bowel disease, antibiotic-associated diarrhea, and Clostridium difficile infection. More recently, the burst landscape has pivoted toward systemic links and, especially, oncology and host immunity: insulin resistance, brain, and periodontal disease appeared as short transitional hotspots, while the strongest current priorities (2022–2025) cluster around *Fusobacterium nucleatum*, tumor microbiome, gastrointestinal cancer, immunity, and activation.

## Discussion

This research analyzed the publications in the field of the Helicobacter pylori–gut microbiota nexus from 2000 to 2025 through a bibliometric analytical method. The Web of Science Core Collection, Scopus, and PubMed were utilized to ensure cross-platform robustness and minimize single-database bias. The annual publication output has witnessed substantial growth, particularly after 2015, and entered a rapid growth phase from 2020 to 2025, reaching a peak in 2022. It indicates that the association between *H. pylori* and the gut microbial ecosystem has become a sustained research hotspot and may still be in an active and robust stage in the following years. The drivers underlying this phenomenon might be attributable to several converging factors. First, the maturation of metagenomic and multi-omics technologies has enabled large-scale characterization of gut microbial composition (49, 62). Second, scientific researchers have made considerable progress in understanding the impact of *H. pylori* eradication on the microbiome, shifting focus from short-term efficacy to broader ecological perturbations (46, 48). This shift delineates a "therapeutic-microecology agenda", emphasizing the critical need to weigh regimen efficacy against microbiome perturbation. Additionally, the post-2018 acceleration in oncology- and immunity-related keywords likely reflects three converging developments: the growing accessibility of multi-omics platforms enabling finer characterization of dysbiotic states in clinical cohorts (30, 31); the demonstration that gut microbial taxa modulate immune checkpoint inhibitor efficacy (28, 58, 59), which stimulated rapid expansion of microbiome-immunotherapy research; and the identification of intratumoral bacterial communities across cancer types (60, 63), which repositioned *H. pylori* within a broader tumor microenvironment framework.

In this research field, China and the United States emerged as the dominant contributors, accounting for a substantial proportion of the total publications. While the United States occupies an important position in international collaborations, China has formed dense and productive domestic research hubs, such as those centered at Capital Medical University and Nanchang University. This concentration of output may be related to disease burden and sustained research investment (2, 61). However, collaboration patterns differ; European countries like the United Kingdom exhibit higher proportions of multi-country publications. Structural barriers such as heterogeneous data-sharing frameworks and variable ethics approval processes increase the "transaction costs" of international harmonization, potentially limiting cross-border partnerships for Asian institutions despite their high domestic output. Therefore, it is of great importance to seek more extensive cross-national collaboration to facilitate a transition from quantity-driven growth toward impact-oriented leadership.

Regarding the publication landscape, research outputs are predominantly published in Q1-ranked journals such as Frontiers in Microbiology, Helicobacter, and Gut Microbes. Interestingly, a structural differentiation by article type was observed. Research-oriented articles were typically produced by larger author teams, consistent with the complexity of multi-omics studies, whereas review articles contained significantly more references. This reflects that high-impact reviews place greater emphasis on comprehensive evidence synthesis and conceptual framework development. Core journals often take up the responsibility of publishing essential studies in the relevant field; thus, authors should align submission strategies with these venues while prioritizing methodological transparency.

One of the primary objectives of this study was to identify the scientific hotspots and evolutionary trajectory of the field. The analysis of keyword co-occurrence and citation bursts revealed three major thematic communities: an infection and antibiotic-resistance axis, a gut ecological dysbiosis axis, and a tumor–immunity axis. Keywords exhibiting the most pronounced post-2018 growth included “dysbiosis,” “immune response,” and “cancer,” reflecting a shift from descriptive microbial profiling toward mechanistic and translational research themes. These temporal changes likely mirror advances in sequencing technologies and growing interest in host–microbiome–immunity interactions. The research frontiers have undergone a progressive shift from baseline microbiome characterization toward post-eradication systemic effects and tumor-resident microbiota (44, 64). Citation burst analysis further confirmed this trajectory, illustrating a paradigm shift from descriptive studies to interventional research and ultimately toward integrative, mechanism-oriented investigations.

From an evolutionary perspective, the intellectual development of research on the *H. pylori*–gut microbiome axis can be described as a progression from foundational metagenomic baselines and mechanistic framing, through an intervention-focused era emphasizing eradication-associated ecological consequences, to the current frontier that centers on tumor-resident microbes and immunotherapy-microbiome crosstalk. The following periodization reflects citation-lagged intellectual influence rather than strict publication chronology. Seminal works frequently achieve peak field-wide impact one to several years after initial publication, as the broader community integrates and builds upon their findings. Each phase is therefore defined by when its landmark contributions became dominant intellectual forces in the field—an approach consistent with the CiteSpace burst detection methodology employed throughout this study.

Phase I — Foundational infrastructure and mechanistic framing (approximately 2010–2015). The early trajectory of this field was shaped by landmark studies in general gut microbiome science that established the methodological infrastructure on which subsequent work depended. Qin et al. (49) assembled the first large-scale human gut microbial gene catalogue from 124 European individuals, providing a reference framework for cross-study functional comparisons. Arumugam et al. (50) reported that gut microbiome composition could be grouped into three robust enterotype clusters independent of geographic origin. The Human Microbiome Project Consortium (62) characterized the healthy human microbiome across multiple body sites, establishing normative baselines against which disease-associated dysbiosis could be measured. Schwabe and Jobin (44) then synthesized evidence linking the microbiota to cancer through immune dysregulation, genotoxicity, and metabolic reprogramming, positioning *H. pylori* as a model of infection-driven carcinogenesis. Applied studies from this period produced heterogeneous findings regarding indirect effects on the distal gut microbiome: some cohort analyses reported minimal perturbation after controlling for diet and geography, while others documented taxon-level shifts and reductions in community richness, exposing limitations related to sample size, sampling site, and methodological variability.

Phase II — Gastric microbiota characterization and the eradication ecology controversy (approximately 2016–2019). The second phase brought direct investigation of the microbiome in *H. pylori*-infected hosts. Aviles-Jimenez et al. (54) and Eun et al. (51) independently documented compositional shifts along the gastritis-to-gastric cancer progression cascade, though with partially discrepant findings at the taxonomic level, raising the unresolved question of whether the non-*H. pylori* microbiota is an active driver or a passive consequence of cancer progression. Ferreira et al. (55) provided evidence favoring the former by demonstrating that gastric microbiota dysbiosis with genotoxic potential was compositionally distinct in gastric cancer patients compared with chronic gastritis cases. Regarding eradication, Yap et al. (56) and Oh et al. (57) reported that antibiotic-based regimens produced detectable perturbations in distal gut communities, including reductions in SCFA-producing taxa. Liou et al. (46) subsequently showed that most such alterations were transient and reversible within weeks to months, though debate about long-term effects in high-risk subgroups continues. Also in this phase, Sivan et al. (59), Vétizou et al. (58), and Routy et al. (28) collectively established the immunotherapy-microbiome axis: the first two demonstrated that specific gut microbial taxa modulated the efficacy of anti-PD-L1 and anti-CTLA-4 therapies respectively, while Routy et al. showed that primary resistance to immune checkpoint inhibitor (ICIs) was linked to dysbiotic gut microbiome composition, with *Akkermansia muciniphila* abundance correlating with treatment response across multiple epithelial tumor types.

Phase III — Intratumoral microbiome and immunotherapy-microbiome crosstalk (approximately 2020–present). Geller et al. (63) showed that intratumor bacteria could mediate chemotherapy resistance through enzymatic drug inactivation, while Nejman et al. (60) established that diverse solid tumors harbor distinct intracellular bacterial communities, dismantling the assumption of the sterile tumor. These findings are situated in *H. pylori* within a broader oncological context. The clinical relevance of microbiome-immunotherapy interactions was reviewed comprehensively by Malfertheiner et al. (6, 24), consolidating evidence that *H. pylori* infection status and microbiome composition influence responses to immune checkpoint inhibitors in gastric cancer. This body of work has generated a central controversy that remains unresolved, as discussed below.

More recently, research has extended into tumor-resident microbiota and systemic host-microbe-immune interactions. Jia et al. (29), in a cohort study led by Lin Shen at Peking University Cancer Hospital, demonstrated that *H. pylori* positivity was associated with significantly longer immune-related progression-free survival in EBV-negative microsatellite-stable gastric cancer patients receiving anti-PD-1/PD-L1 therapy. Routy et al. (65) subsequently demonstrated in a phase I trial that fecal microbiota transplantation from healthy donors combined with anti-PD-1 therapy was safe and achieved a 65% objective response rate in advanced melanoma patients, providing clinical proof-of-concept that gut microbiome modulation can enhance systemic ICI outcomes. The central controversy concerns whether *H. pylori* infection promotes a pro-inflammatory, ICI-responsive tumor microenvironment or an immune-excluded, ICI-resistant state. *H. pylori* positivity has been associated with favorable immune measures in some gastric cancer cohorts, yet in extra-gastric settings including colorectal cancer, melanoma, and lung cancer, infection has correlated with poorer survival or reduced ICI efficacy (66). Alongside these immunological questions, Chen et al. (20) highlighted that *H. pylori* eradication itself reshapes the gut microbial community in ways that may have downstream consequences for host immune tone. Resolving these associations will require larger geographically diverse cohorts, standardized longitudinal sampling, shotgun metagenomics, spatial microbial mapping, and integrated immune and metabolite profiling to determine when *H. pylori*-associated dysbiosis is protective, deleterious, or neutral in specific oncologic contexts.

Given the exponential growth of high-dimensional data, the field is witnessing a computational turn. Future research directions will likely demand longitudinal studies with follow-up periods exceeding 12 months to characterize long-term trajectories of microbiome recovery (46). Furthermore, advanced causal inference methods (e.g., Mendelian randomization) should be employed to move beyond mere associations (67). Greater attention must be paid to population heterogeneity, particularly regarding regional environmental factors and host genetics. Crucially, artificial intelligence and machine learning algorithms should be leveraged to decipher complex multi-omics networks and identify a therapeutic "Pareto front" that maximizes eradication rates while minimizing microecological disruption (68, 69). Finally, standardized protocols co-collecting stomach and distal-gut metagenomes, viromes, and host mucosal transcriptomes are required to build comprehensive interaction networks.

This review is subject to several limitations. First, as a bibliometric analysis, data collection and processing highly depend on software. Although parallel analyses across multiple databases (WoS, Scopus, PubMed) were conducted to enhance reproducibility, the reliance on title and keyword-based retrieval may have excluded relevant studies not explicitly labeled as microbiome research. Secondly, only articles written in English were collected, which means some valuable studies in other languages may be missed. Thirdly, since delay exists in citation impact, some high-quality studies recently published may be underestimated on their impact. Despite all these, this study will help researchers to understand the developing trend, hotspots, and frontiers in the field of the *H. pylori*–gut microbiota nexus (70).

## Conclusion

In conclusion, the research on the *H. pylori*–gut microbiota nexus has entered a rapid growth phase and remains a robust hotspot. Key priorities for future research directions include: 1. Mechanisms underlying the "therapeutic-microecology" balance; 2. AI-driven precision strategies for *H. pylori* eradication; 3. Crosstalk between *H. pylori* and cancer immunotherapy. With these insights, researchers are well-equipped to translate ecological knowledge into clinical precision.

## Data Availability

The original contributions presented in the study are included in the article/**Supplementary Material**. Further inquiries can be directed to the corresponding authors.

## Supplementary material

Supplementary Figure 1 Global publication trends and geographical distribution based on Scopus data. **(A)** Annual and accumulated publication output (2001–2025). **(B)** World map visualization of country scientific production.

Supplementary Figure 2 Global publication trends and geographical distribution based on PubMed data. **(A)** Annual and accumulated publication output (2000–2025). **(B)** World map visualization of country scientific production.

Supplementary Figure 3 Article types and authorship characteristics based on Scopus data. **(A)** Cumulative publication trends of research articles and reviews. **(B)** Comparison of author numbers between document types. **(C, D)** Distribution of author numbers stratified by journal impact factor groups for research articles **(C)** and reviews **(D)**.

Supplementary Figure 4 Research output and impact of top countries and institutions based on Scopus data. **(A)** Publication volume, international collaboration, and citation impact of the top 10 productive countries. **(B)** Total publication output and temporal accumulation trends of the top 10 affiliations.

Supplementary Figure 5 Research output and impact of top countries and institutions based on PubMed data. **(A)** Publication volume and international collaboration of the top 10 productive countries. **(B)** Total publication output and temporal accumulation trends of the top 10 affiliations.

Supplementary Figure 6 Journal distribution and publication dynamics based on Scopus data. **(A)** The top 10 most productive journals. Bars represent publication volume (color-coded by JCR quartile), while the line indicates the Impact Factor (shapes denote the publisher’s country). **(B)** Annual publication growth trends of the top 10 journals.

Supplementary Figure 7 Journal distribution and publication dynamics based on PubMed data. **(A)** The most productive journals. Bars represent publication volume (color-coded by JCR quartile), while the line indicates the Impact Factor (shapes denote the publisher’s country). **(B)** Annual publication growth trends of the top journals.

Supplementary Figure 8 Author collaboration networks across databases. Network visualization of author collaborations based on Web of Science data **(A)**, Scopus data **(B)** and PubMed data **(C)**.

Supplementary Figure 9 Scientific production and impact of top authors based on Scopus data. **(A)** Relationship between the number of articles and local H-index. **(B)** Annual publication output and citation intensity of leading contributors.

Supplementary Figure 10 Top 25 references with the strongest citation bursts.

Supplementary Figure 11 Keyword frequency and temporal trends analysis based on Scopus data. **(A)** Distribution of high-frequency keywords. **(B)** Accumulated growth trends of top keywords over time.

Supplementary Figure 12 Three-field plot of countries, institutions, and keywords.

Supplementary Figure 13 Co-occurrence analysis and visualization of keywords.

Supplementary Figure 14 Top keywords with the strongest citation bursts.

## References

[1] Chmiela M Karwowska Z Gonciarz W Allushi B Stączek P . Host pathogen interactions in Helicobacter pylori related gastric cancer. World J Gastroenterol. (2017) 23:1521–40. doi: 10.3748/wjg.v23.i9.1521. PMID: 28321154 PMC5340805

[2] Hooi JKY Lai WY Ng WK Suen MMY Underwood FE Tanyingoh D . Global prevalence of Helicobacter pylori infection: Systematic review and meta-analysis. Gastroenterology. (2017) 153:420–9. doi: 10.1053/j.gastro.2017.04.022. PMID: 28456631

[3] Brown LM . Helicobacter pylori: epidemiology and routes of transmission. Epidemiol Rev. (2000) 22:283–97. doi: 10.1093/oxfordjournals.epirev.a018040. PMID: 11218379

[4] Asaka M Kudo M Kato M Sugiyama T Takeda H . Review article: Long-term Helicobacter pylori infection--from gastritis to gastric cancer. Aliment Pharmacol Ther. (1998) 12:9–15. doi: 10.1111/j.1365-2036.1998.00007.x. PMID: 9701000

[5] Umar Z Tang J-W Marshall BJ Tay ACY Wang L . Rapid diagnosis and precision treatment of Helicobacter pylori infection in clinical settings. Crit Rev Microbiol. (2025) 51:369–98. doi: 10.1080/1040841X.2024.2364194. PMID: 38910506

[6] Malfertheiner P Camargo MC El-Omar E Liou J-M Peek R Schulz C . Helicobacter pylori infection. Nat Rev Dis Primer. (2023) 9:19. doi: 10.1038/s41572-023-00431-8. PMID: 37081005 PMC11558793

[7] Camilo V Sugiyama T Touati E . Pathogenesis of Helicobacter pylori infection. Helicobacter. (2017) 22(Suppl. 1):e12405. doi: 10.1111/hel.12405. PMID: 28891130

[8] Pickard JM Zeng MY Caruso R Núñez G . Gut microbiota: Role in pathogen colonization, immune responses, and inflammatory disease. Immunol Rev. (2017) 279:70–89. doi: 10.1111/imr.12567. PMID: 28856738 PMC5657496

[9] Boulangé CL Neves AL Chilloux J Nicholson JK Dumas M-E . Impact of the gut microbiota on inflammation, obesity, and metabolic disease. Genome Med. (2016) 8:42. doi: 10.1186/s13073-016-0303-2. PMID: 27098727 PMC4839080

[10] Ma X Brinker E Graff EC Cao W Gross AL Johnson AK . Whole-genome shotgun metagenomic sequencing reveals distinct gut microbiome signatures of obese cats. Microbiol Spectr. (2022) 10:e0083722. doi: 10.1128/spectrum.00837-22. PMID: 35467389 PMC9241680

[11] Haley KP Gaddy JA . Nutrition and Helicobacter pylori: Host diet and nutritional immunity influence bacterial virulence and disease outcome. Gastroenterol Res Pract. (2016) 2016:3019362. doi: 10.1155/2016/3019362. PMID: 27688750 PMC5027306

[12] Mahdavi J Sondén B Hurtig M Olfat FO Forsberg L Roche N . Helicobacter pylori SabA adhesin in persistent infection and chronic inflammation. Science. (2002) 297:573–8. doi: 10.1126/science.1069076. PMID: 12142529 PMC2570540

[13] Mégraud F . Toxic factors of Helicobacter pylori. Eur J Gastroenterol Hepatol. (1994) 6:S5–10. 7735936

[14] Pope AJ Toseland CD Rushant B Richardson S McVey M Hills J . Effect of potent urease inhibitor, fluorofamide, on Helicobacter sp. *in vivo* and *in vitro*. Dig Dis Sci. (1998) 43:109–19. doi: 10.1023/a:1018884322973. PMID: 9508511

[15] Deng ZH Li X Liu L Zeng HM Chen BF Peng J . Role of gut microbiota and Helicobacter pylori in inflammatory bowel disease through immune-mediated synergistic actions. World J Gastroenterol. (2024) 30:5097–103. doi: 10.3748/wjg.v30.i47.5097. PMID: 39713161 PMC11612865

[16] Dooyema SDR Noto JM Wroblewski LE Piazuelo MB Krishna U Suarez G . Helicobacter pylori actively suppresses innate immune nucleic acid receptors. Gut Microbes. (2022) 14:2105102. doi: 10.1080/19490976.2022.2105102. PMID: 35905376 PMC9341374

[17] Karkhah A Ebrahimpour S Rostamtabar M Koppolu V Darvish S Vasigala VKR . Helicobacter pylori evasion strategies of the host innate and adaptive immune responses to survive and develop gastrointestinal diseases. Microbiol Res. (2019) 218:49–57. doi: 10.1016/j.micres.2018.09.011. PMID: 30454658

[18] Mohammadi SO Yadegar A Kargar M Mirjalali H Kafilzadeh F . The impact of Helicobacter pylori infection on gut microbiota-endocrine system axis; modulation of metabolic hormone levels and energy homeostasis. J Diabetes Metab Disord. (2020) 19:1855–61. doi: 10.1007/s40200-020-00608-y. PMID: 33553045 PMC7843871

[19] Morales-Marroquin E Hanson B Greathouse L de la Cruz-Munoz N Messiah SE . Comparison of methodological approaches to human gut microbiota changes in response to metabolic and bariatric surgery: A systematic review. Obes Rev Off J Int Assoc Study Obes. (2020) 21:e13025. doi: 10.1111/obr.13025. PMID: 32249534

[20] Chen C-C Liou J-M Lee Y-C Hong T-C El-Omar EM Wu M-S . The interplay between Helicobacter pylori and gastrointestinal microbiota. Gut Microbes. (2021) 13:1–22. doi: 10.1080/19490976.2021.1909459. PMID: 33938378 PMC8096336

[21] Espinoza JL Matsumoto A Tanaka H Matsumura I . Gastric microbiota: An emerging player in Helicobacter pylori-induced gastric Malignancies. Cancer Lett. (2018) 414:147–52. doi: 10.1016/j.canlet.2017.11.009. PMID: 29138097

[22] Engelsberger V Gerhard M Mejías-Luque R . Effects of Helicobacter pylori infection on intestinal microbiota, immunity and colorectal cancer risk. Front Cell Infect Microbiol. (2024) 14:1339750. doi: 10.3389/fcimb.2024.1339750. PMID: 38343887 PMC10853882

[23] Francescone R Hou V Grivennikov SI . Microbiome, inflammation, and cancer. Cancer J. (2014) 20:181–9. doi: 10.1097/PPO.0000000000000048. PMID: 24855005 PMC4112188

[24] Malfertheiner P Megraud F Rokkas T Gisbert JP Liou J-M Schulz C . Management of Helicobacter pylori infection: the Maastricht VI/Florence consensus report. Gut. (2022) 71:gutjnl–2022-327745. doi: 10.1136/gutjnl-2022-327745. PMID: 35944925

[25] Testerman TL Morris J . Beyond the stomach: an updated view of Helicobacter pylori pathogenesis, diagnosis, and treatment. World J Gastroenterol. (2014) 20:12781–808. doi: 10.3748/wjg.v20.i36.12781. PMID: 25278678 PMC4177463

[26] Eroğlu Haktanır A Çelebi A . Effects of intragastric Helicobacter pylori distribution on clinical presentation, upper gastrointestinal endoscopy, esophageal manometry, and pH-impedance metrics. J Clin Med. (2025) 14:6818. doi: 10.3390/jcm14196818. PMID: 41095895 PMC12524794

[27] Deng R Zheng H Cai H Li M Shi Y Ding S . Effects of helicobacter pylori on tumor microenvironment and immunotherapy responses. Front Immunol. (2022) 13:923477. doi: 10.3389/fimmu.2022.923477. PMID: 35967444 PMC9371381

[28] Routy B Le Chatelier E Derosa L Duong CPM Alou MT Daillère R . Gut microbiome influences efficacy of PD-1-based immunotherapy against epithelial tumors. Science. (2018) 359:91–7. doi: 10.1126/science.aan3706. PMID: 29097494

[29] Jia K Chen Y Xie Y Wang X Hu Y Sun Y . Helicobacter pylori and immunotherapy for gastrointestinal cancer. Innov Camb Mass. (2024) 5:100561. doi: 10.1016/j.xinn.2023.100561. PMID: 38379784 PMC10878118

[30] Chetty A Blekhman R . Multi-omic approaches for host-microbiome data integration. Gut Microbes. (2024) 16:2297860. doi: 10.1080/19490976.2023.2297860. PMID: 38166610 PMC10766395

[31] Daliri E-M Ofosu FK Chelliah R Lee BH Oh D-H . Challenges and perspective in integrated multi-omics in gut microbiota studies. Biomolecules. (2021) 11:300. doi: 10.3390/biom11020300. PMID: 33671370 PMC7922017

[32] Bolyen E Rideout JR Dillon MR Bokulich NA Abnet CC Al-Ghalith GA . Reproducible, interactive, scalable and extensible microbiome data science using QIIME 2. Nat Biotechnol. (2019) 37:852–7. doi: 10.1038/s41587-019-0209-9. PMID: 31341288 PMC7015180

[33] Yang C Mai J Cao X Burberry A Cominelli F Zhang L . ggpicrust2: an R package for PICRUSt2 predicted functional profile analysis and visualization. Bioinforma Oxf Engl. (2023) 39:btad470. doi: 10.1093/bioinformatics/btad470. PMID: 37527009 PMC10425198

[34] Franzosa EA McIver LJ Rahnavard G Thompson LR Schirmer M Weingart G . Species-level functional profiling of metagenomes and metatranscriptomes. Nat Methods. (2018) 15:962–8. doi: 10.1038/s41592-018-0176-y. PMID: 30377376 PMC6235447

[35] Cantalapiedra CP Hernández-Plaza A Letunic I Bork P Huerta-Cepas J . eggNOG-mapper v2: Functional annotation, orthology assignments, and domain prediction at the metagenomic scale. Mol Biol Evol. (2021) 38:5825–9. doi: 10.1093/molbev/msab293. PMID: 34597405 PMC8662613

[36] Liu D Zhu J Ma X Zhang L Wu Y Zhu W . Transcriptomic and metabolomic profiling in Helicobacter pylori-induced gastric cancer identified prognosis- and immunotherapy-relevant gene signatures. Front Cell Dev Biol. (2021) 9:769409. doi: 10.3389/fcell.2021.769409. PMID: 35004676 PMC8740065

[37] White B Sterrett JD Grigoryan Z Lally L Heinze JD Alikhan H . Characterization of gut microbiome and metabolome in Helicobacter pylori patients in an underprivileged community in the United States. World J Gastroenterol. (2021) 27:5575–94. doi: 10.3748/wjg.v27.i33.5575. PMID: 34588753 PMC8433610

[38] Zaramella A Arcidiacono D Duci M Benna C Pucciarelli S Fantin A . Predictive value of a gastric microbiota dysbiosis test for stratifying cancer risk in atrophic gastritis patients. Nutrients. (2024) 17:142. doi: 10.3390/nu17010142. PMID: 39796578 PMC11722812

[39] Salahi-Niri A Nabavi-Rad A Monaghan TM Rokkas T Doulberis M Sadeghi A . Global prevalence of Helicobacter pylori antibiotic resistance among children in the World Health Organization regions between 2000 and 2023: a systematic review and meta-analysis. BMC Med. (2024) 22:598. doi: 10.1186/s12916-024-03816-y. PMID: 39710669 PMC11664859

[40] Hibstu Z Belew H Akelew Y Mengist HM . Phage therapy: A different approach to fight bacterial infections. Biol Targets Ther. (2022) 16:173–86. doi: 10.2147/BTT.S381237. PMID: 36225325 PMC9550173

[41] Muñoz AB Stepanian J Trespalacios AA Vale FF . Bacteriophages of Helicobacter pylori. Front Microbiol. (2020) 11:549084. doi: 10.3389/fmicb.2020.549084. PMID: 33281754 PMC7688985

[42] Uchiyama J Takeuchi H Kato S Gamoh K Takemura-Uchiyama I Ujihara T . Characterization of Helicobacter pylori bacteriophage KHP30. Appl Environ Microbiol. (2013) 79:3176–84. doi: 10.1128/AEM.03530-12. PMID: 23475617 PMC3685256

[43] TITAN Group . Transparency in the reporting of artificial intelligence – the TITAN guideline - Premier Science. Prem J Sci. (2025) 10:100082. doi: 10.70389/PJS.100082

[44] Schwabe RF Jobin C . The microbiome and cancer. Nat Rev Cancer. (2013) 13:800–12. doi: 10.1038/nrc3610. PMID: 24132111 PMC3986062

[45] Lertpiriyapong K Whary MT Muthupalani S Lofgren JL Gamazon ER Feng Y . Gastric colonisation with a restricted commensal microbiota replicates the promotion of neoplastic lesions by diverse intestinal microbiota in the Helicobacter pylori INS-GAS mouse model of gastric carcinogenesis. Gut. (2014) 63:54–63. doi: 10.1136/gutjnl-2013-305178. PMID: 23812323 PMC4023484

[46] Liou J-M Chen C-C Chang C-M Fang Y-J Bair M-J Chen P-Y . Long-term changes of gut microbiota, antibiotic resistance, and metabolic parameters after Helicobacter pylori eradication: a multicentre, open-label, randomised trial. Lancet Infect Dis. (2019) 19:1109–20. doi: 10.1016/S1473-3099(19)30272-5. PMID: 31559966

[47] Chen L Xu W Lee A He J Huang B Zheng W . The impact of Helicobacter pylori infection, eradication therapy and probiotic supplementation on gut microenvironment homeostasis: An open-label, randomized clinical trial. EBioMedicine. (2018) 35:87–96. doi: 10.1016/j.ebiom.2018.08.028. PMID: 30145102 PMC6161473

[48] Jakobsson HE Jernberg C Andersson AF Sjölund-Karlsson M Jansson JK Engstrand L . Short-term antibiotic treatment has differing long-term impacts on the human throat and gut microbiome. PloS One. (2010) 5:e9836. doi: 10.1371/journal.pone.0009836. PMID: 20352091 PMC2844414

[49] Qin J Li R Raes J Arumugam M Burgdorf KS Manichanh C . A human gut microbial gene catalogue established by metagenomic sequencing. Nature. (2010) 464:59–65. doi: 10.1038/nature08821. PMID: 20203603 PMC3779803

[50] Arumugam M Raes J Pelletier E Le Paslier D Yamada T Mende DR . Enterotypes of the human gut microbiome. Nature. (2011) 473:174–80. doi: 10.1038/nature09944. PMID: 21508958 PMC3728647

[51] Eun CS Kim BK Han DS Kim SY Kim KM Choi BY . Differences in gastric mucosal microbiota profiling in patients with chronic gastritis, intestinal metaplasia, and gastric cancer using pyrosequencing methods. Helicobacter. (2014) 19:407–16. doi: 10.1111/hel.12145. PMID: 25052961

[52] Arthur JC Perez-Chanona E Mühlbauer M Tomkovich S Uronis JM Fan T-J . Intestinal inflammation targets cancer-inducing activity of the microbiota. Science. (2012) 338:120–3. doi: 10.1126/science.1224820. PMID: 22903521 PMC3645302

[53] Kostic AD Chun E Robertson L Glickman JN Gallini CA Michaud M . Fusobacterium nucleatum potentiates intestinal tumorigenesis and modulates the tumor-immune microenvironment. Cell Host Microbe. (2013) 14:207–15. doi: 10.1016/j.chom.2013.07.007. PMID: 23954159 PMC3772512

[54] Aviles-Jimenez F Vazquez-Jimenez F Medrano-Guzman R Mantilla A Torres J . Stomach microbiota composition varies between patients with non-atrophic gastritis and patients with intestinal type of gastric cancer. Sci Rep. (2014) 4:4202. doi: 10.1038/srep04202. PMID: 24569566 PMC3935187

[55] Ferreira RM Pereira-Marques J Pinto-Ribeiro I Costa JL Carneiro F MaChado JC . Gastric microbial community profiling reveals a dysbiotic cancer-associated microbiota. Gut. (2018) 67:226–36. doi: 10.1136/gutjnl-2017-314205. PMID: 29102920 PMC5868293

[56] Yap T-C Gan H-M Lee Y-P Leow A-R Azmi AN Francois F . Helicobacter pylori eradication causes perturbation of the human gut microbiome in young adults. PloS One. (2016) 11:e0151893. doi: 10.1371/journal.pone.0151893. PMID: 26991500 PMC4798770

[57] Oh B Kim B-S Kim JW Kim JS Koh S-J Kim BG . The effect of probiotics on gut microbiota during the Helicobacter pylori eradication: Randomized controlled trial. Helicobacter. (2016) 21:165–74. doi: 10.1111/hel.12270. PMID: 26395781

[58] Vétizou M Pitt JM Daillère R Lepage P Waldschmitt N Flament C . Anticancer immunotherapy by CTLA-4 blockade relies on the gut microbiota. Science. (2015) 350:1079–84. doi: 10.1126/science.aad1329. PMID: 26541610 PMC4721659

[59] Sivan A Corrales L Hubert N Williams JB Aquino-Michaels K Earley ZM . Commensal Bifidobacterium promotes antitumor immunity and facilitates anti-PD-L1 efficacy. Science. (2015) 350:1084–9. doi: 10.1126/science.aac4255. PMID: 26541606 PMC4873287

[60] Nejman D Livyatan I Fuks G Gavert N Zwang Y Geller LT . The human tumor microbiome is composed of tumor type-specific intracellular bacteria. Science. (2020) 368:973–80. doi: 10.1126/science.aay9189. PMID: 32467386 PMC7757858

[61] Sung H Ferlay J Siegel RL Laversanne M Soerjomataram I Jemal A . Global cancer statistics 2020: GLOBOCAN estimates of incidence and mortality worldwide for 36 cancers in 185 countries. CA Cancer J Clin. (2021) 71:209–49. doi: 10.3322/caac.21660. PMID: 33538338

[62] Human Microbiome Project Consortium . Structure, function and diversity of the healthy human microbiome. Nature. (2012) 486:207–14. doi: 10.1038/nature11234. PMID: 22699609 PMC3564958

[63] Geller LT Barzily-Rokni M Danino T Jonas OH Shental N Nejman D . Potential role of intratumor bacteria in mediating tumor resistance to the chemotherapeutic drug gemcitabine. Science. (2017) 357:1156–63. doi: 10.1126/science.aah5043. PMID: 28912244 PMC5727343

[64] Sepich-Poore GD Zitvogel L Straussman R Hasty J Wargo JA Knight R . The microbiome and human cancer. Science. (2021) 371:eabc4552. doi: 10.1126/science.abc4552. PMID: 33766858 PMC8767999

[65] Routy B Lenehan JG Miller WH Jamal R Messaoudene M Daisley BA . Fecal microbiota transplantation plus anti-PD-1 immunotherapy in advanced melanoma: a phase I trial. Nat Med. (2023) 29:2121–32. doi: 10.1038/s41591-023-02453-x. PMID: 37414899

[66] Hou K Wu Z-X Chen X-Y Wang J-Q Zhang D Xiao C . Microbiota in health and diseases. Signal Transduct Target Ther. (2022) 7:135. doi: 10.1038/s41392-022-00974-4. PMID: 35461318 PMC9034083

[67] Kurilshikov A Medina-Gomez C Bacigalupe R Radjabzadeh D Wang J Demirkan A . Large-scale association analyses identify host factors influencing human gut microbiome composition. Nat Genet. (2021) 53:156–65. doi: 10.1038/s41588-020-00763-1. PMID: 33462485 PMC8515199

[68] Marcos-Zambrano LJ Karaduzovic-Hadziabdic K Loncar Turukalo T Przymus P Trajkovik V Aasmets O . Applications of machine learning in human microbiome studies: A review on feature selection, biomarker identification, disease prediction and treatment. Front Microbiol. (2021) 12:634511. doi: 10.3389/fmicb.2021.634511. PMID: 33737920 PMC7962872

[69] Li L Liu Z Qin J Xiong G Yang C Cai F . Constructing inflammatory bowel disease diagnostic models based on k-mer and machine learning. Front Microbiol. (2025) 16:1578005. doi: 10.3389/fmicb.2025.1578005. PMID: 40636504 PMC12239758

[70] Donthu N Kumar S Mukherjee D Pandey N Lim WM . How to conduct a bibliometric analysis: An overview and guidelines. J Bus Res. (2021) 133:285–96. doi: 10.1016/j.jbusres.2021.04.070. PMID: 41872344

