# Supplementary figures and images for: Worldwide research trends on the Helicobacter pylori–gut microbiome nexus: a bibliometric analysis
Source: Front Immunol. 2026 Apr 16;17:1794021. doi: 10.3389/fimmu.2026.1794021 (PMC13128404; doi:10.3389/fimmu.2026.1794021)

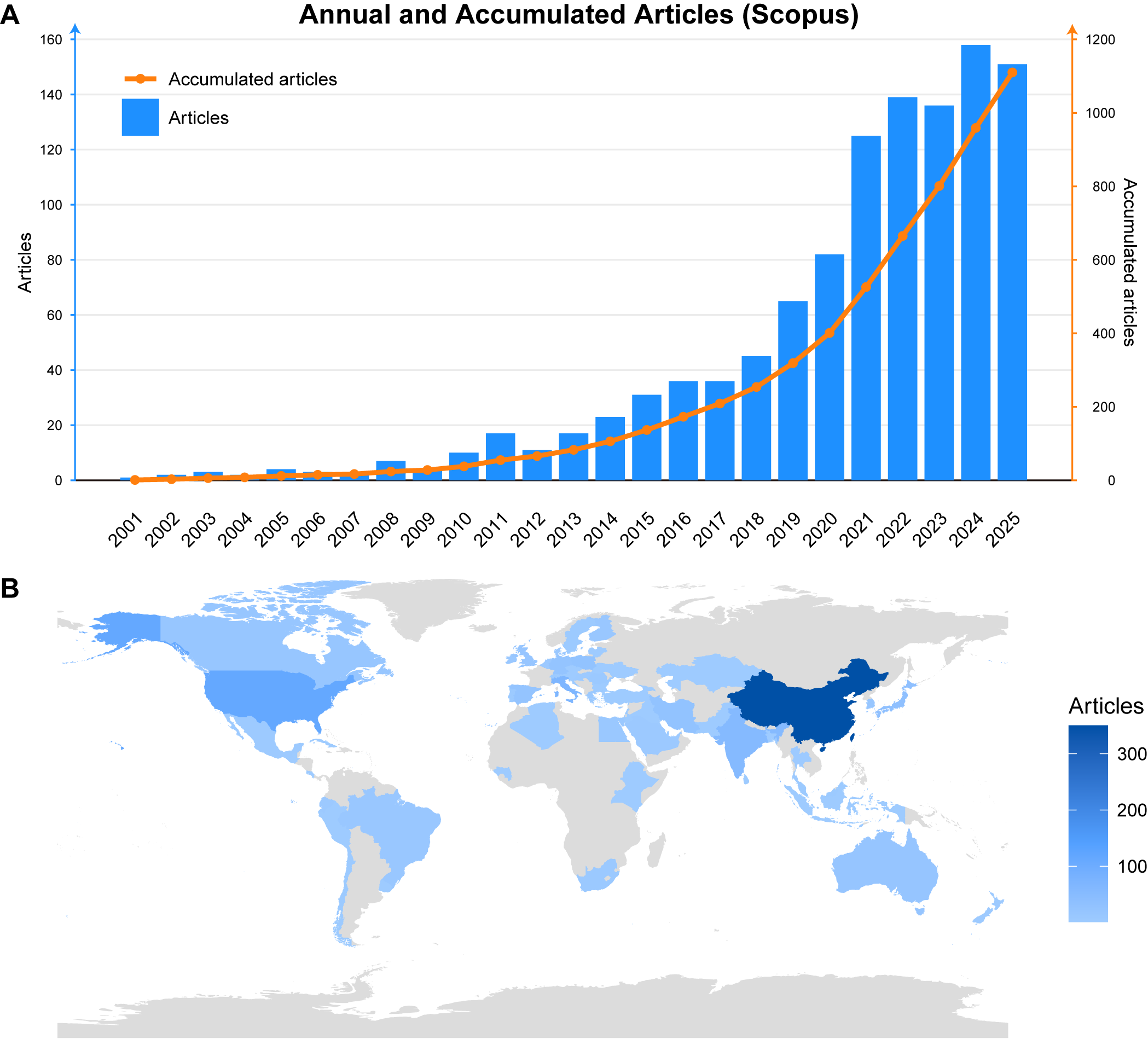

Supplement: Supplementary Figure 1 — Global publication trends and geographical distribution based on Scopus data. (A) Annual and accumulated publication output (2001–2025). (B) World map visualization of country scientific production. [file Image1.tif]

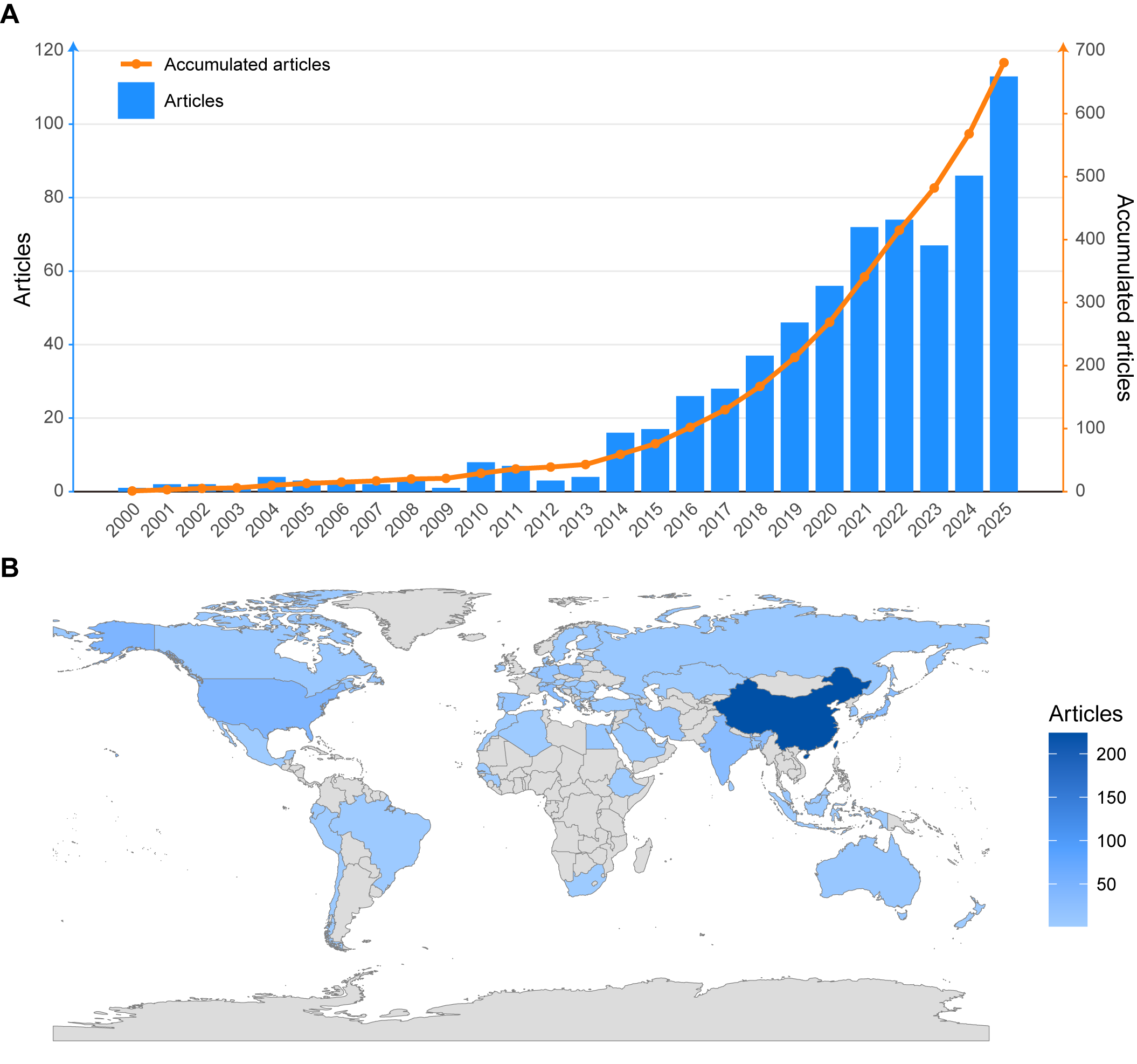

Supplement: Supplementary Figure 2 — Global publication trends and geographical distribution based on PubMed data. (A) Annual and accumulated publication output (2000–2025). (B) World map visualization of country scientific production. [file Image2.tif]

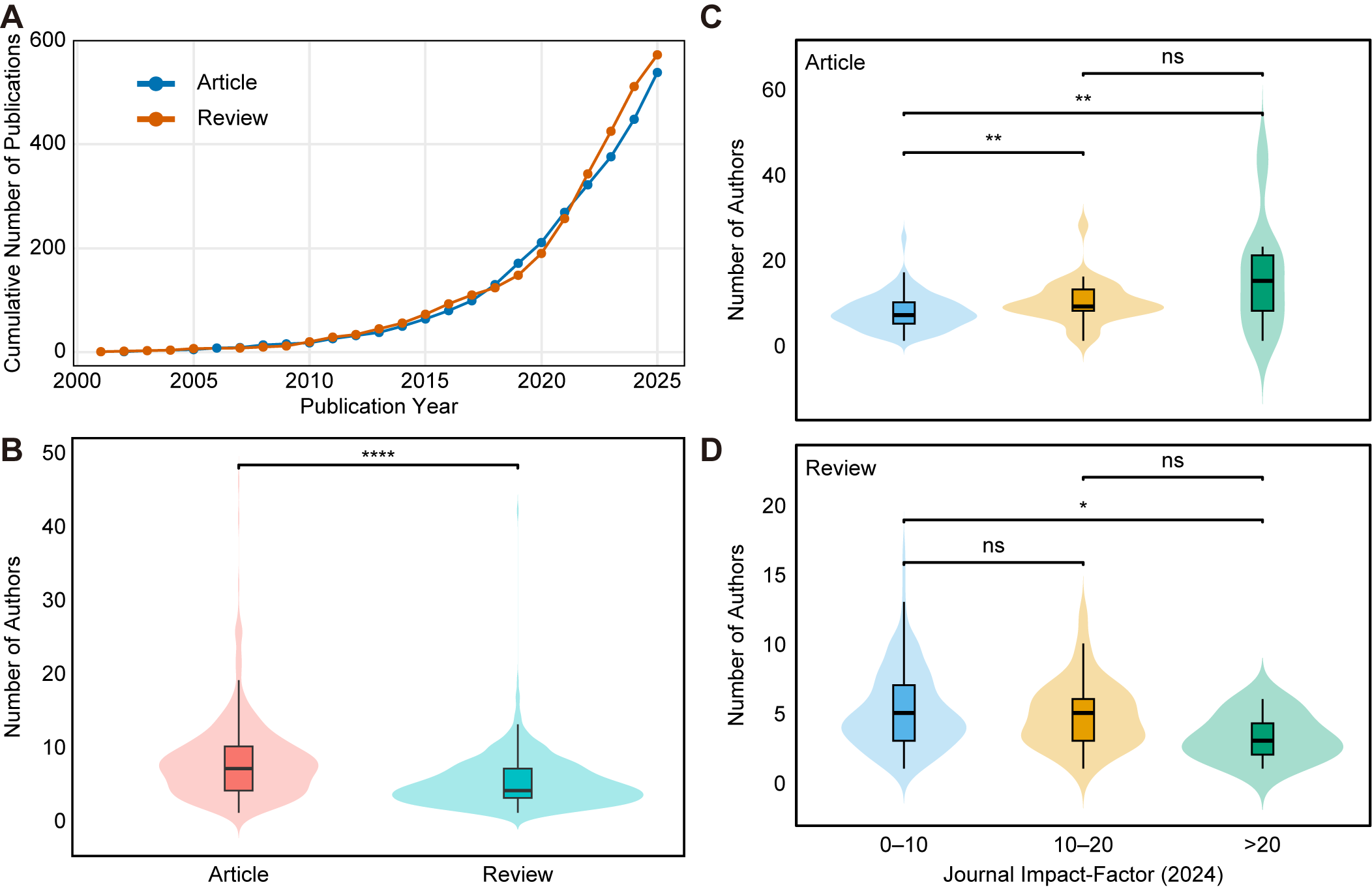

Supplement: Supplementary Figure 3 — Article types and authorship characteristics based on Scopus data. (A) Cumulative publication trends of research articles and reviews. (B) Comparison of author numbers between document types. (C, D) Distribution of author numbers stratified by journal impact factor groups for research articles (C) and reviews (D). [file Image3.tif]

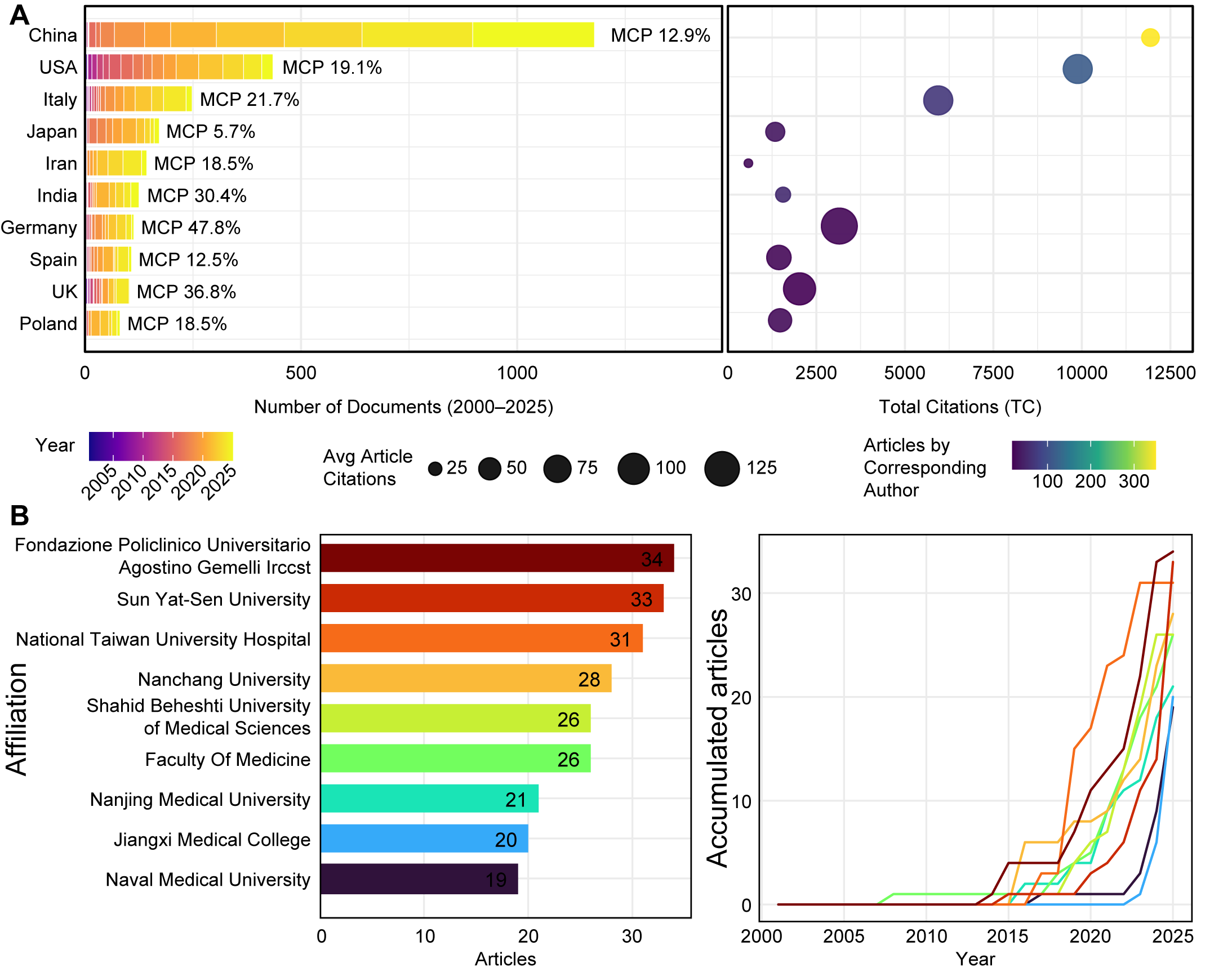

Supplement: Supplementary Figure 4 — Research output and impact of top countries and institutions based on Scopus data. (A) Publication volume, international collaboration, and citation impact of the top 10 productive countries. (B) Total publication output and temporal accumulation trends of the top 10 affiliations. [file Image4.tif]

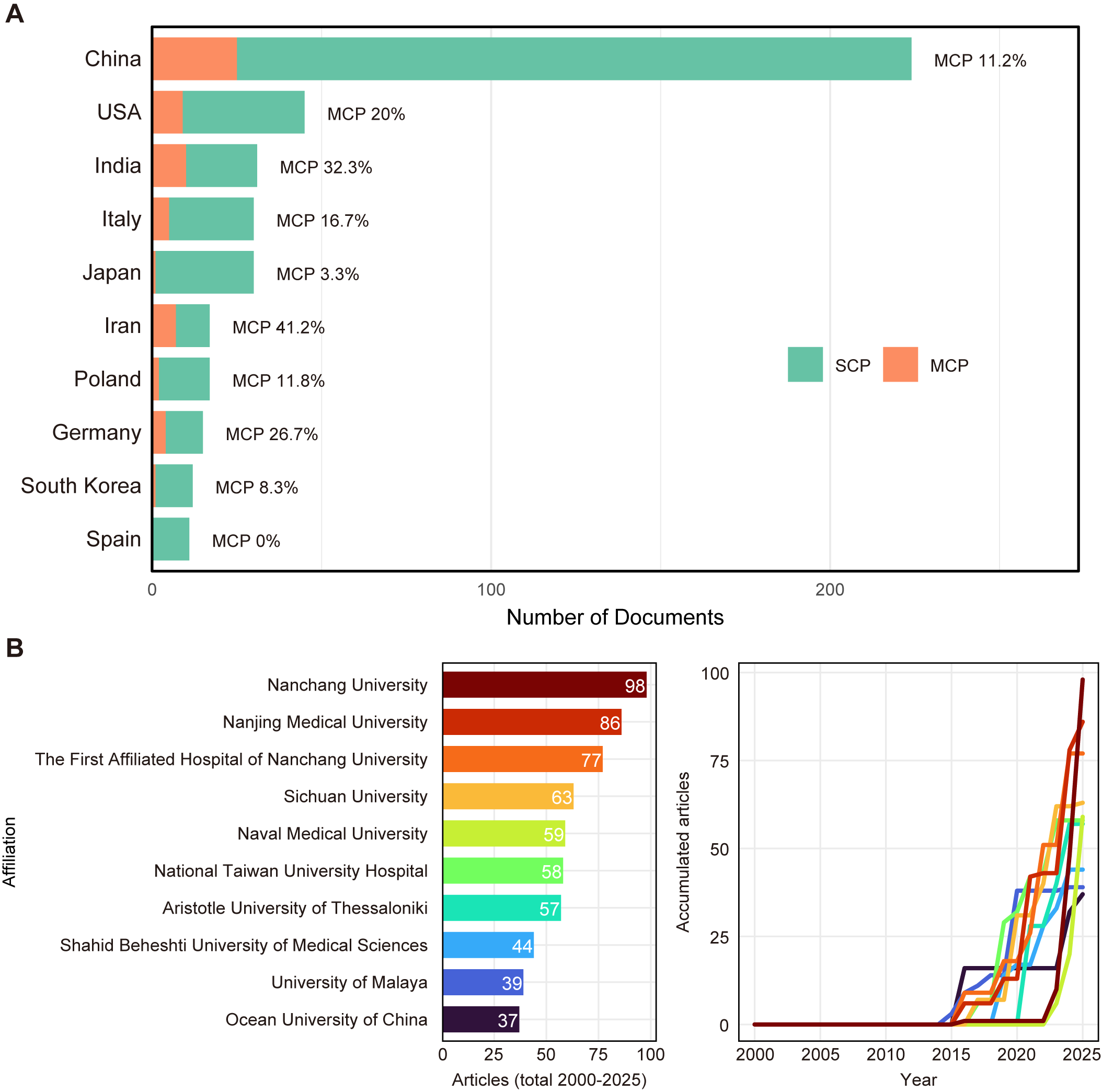

Supplement: Supplementary Figure 5 — Research output and impact of top countries and institutions based on PubMed data. (A) Publication volume and international collaboration of the top 10 productive countries. (B) Total publication output and temporal accumulation trends of the top 10 affiliations. [file Image5.tif]

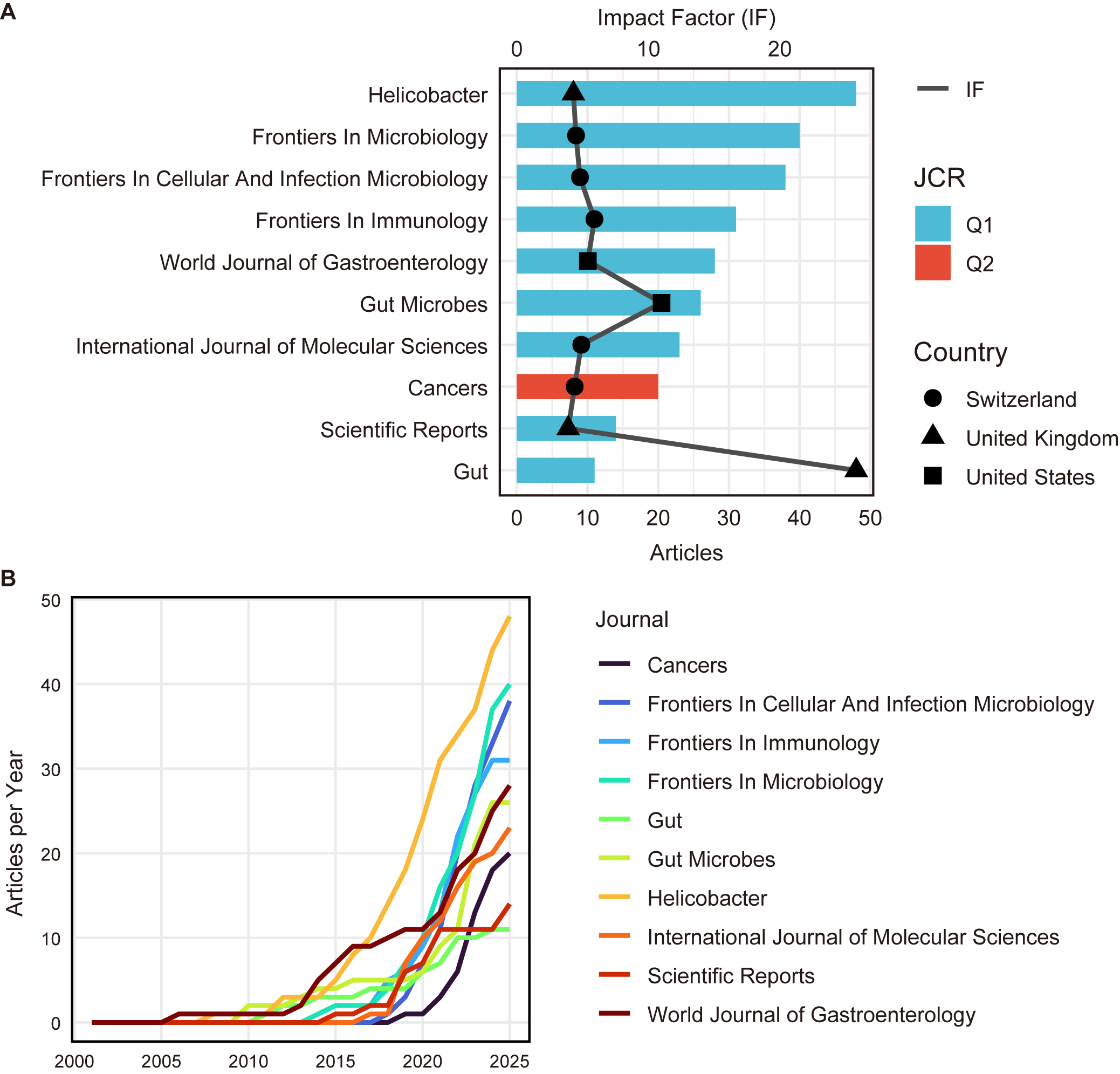

Supplement: Supplementary Figure 6 — Journal distribution and publication dynamics based on Scopus data. (A) The top 10 most productive journals. Bars represent publication volume (color-coded by JCR quartile), while the line indicates the Impact Factor (shapes denote the publisher’s country). (B) Annual publication growth trends of the top 10 journals. [file Image6.tif]

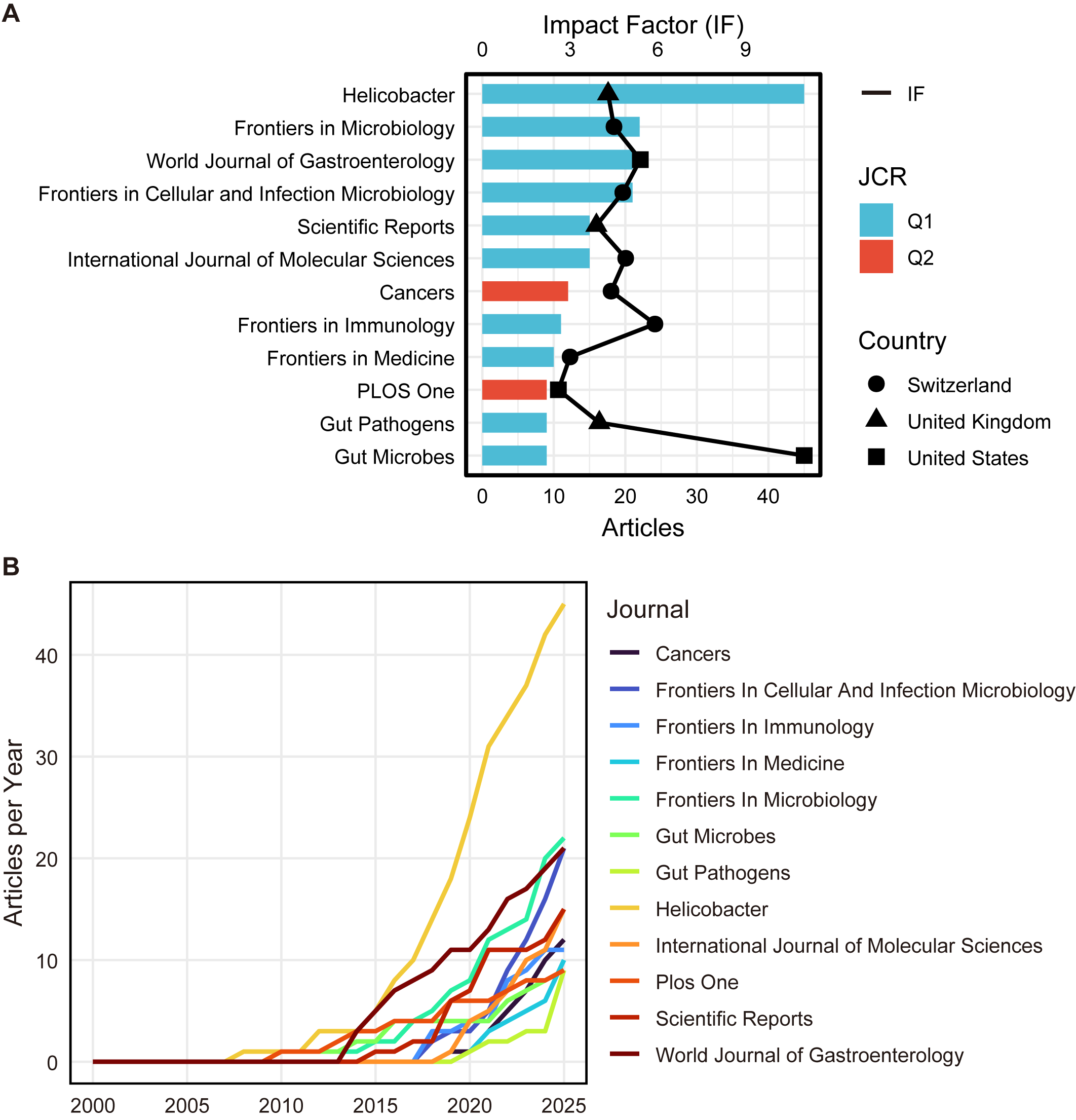

Supplement: Supplementary Figure 7 — Journal distribution and publication dynamics based on PubMed data. (A) The most productive journals. Bars represent publication volume (color-coded by JCR quartile), while the line indicates the Impact Factor (shapes denote the publisher’s country). (B) Annual publication growth trends of the top journals. [file Image7.tif]

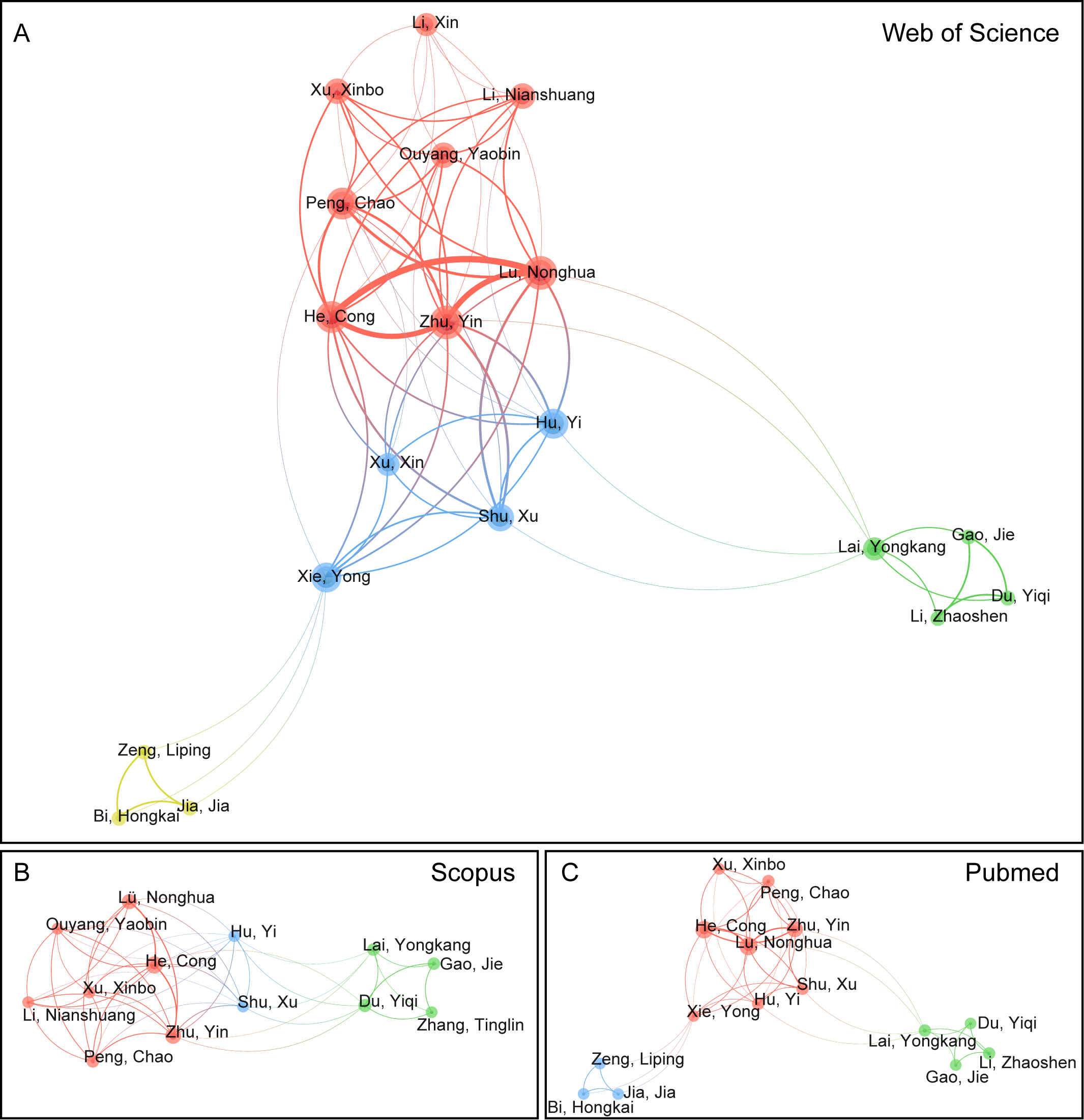

Supplement: Supplementary Figure 8 — Author collaboration networks across databases. Network visualization of author collaborations based on Web of Science data (A), Scopus data (B) and PubMed data (C). [file Image8.tif]

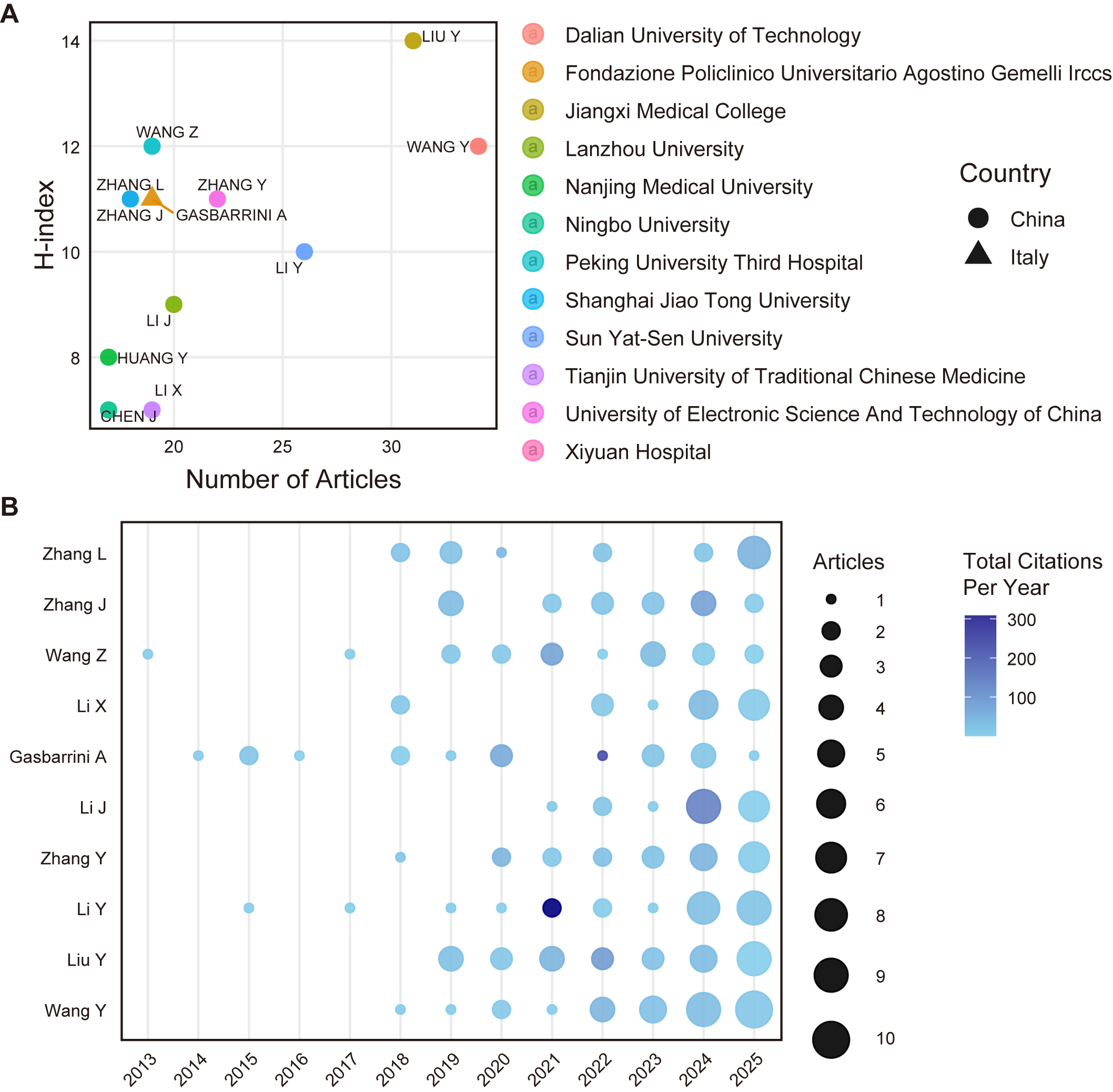

Supplement: Supplementary Figure 9 — Scientific production and impact of top authors based on Scopus data. (A) Relationship between the number of articles and local H-index. (B) Annual publication output and citation intensity of leading contributors. [file Image9.tif]

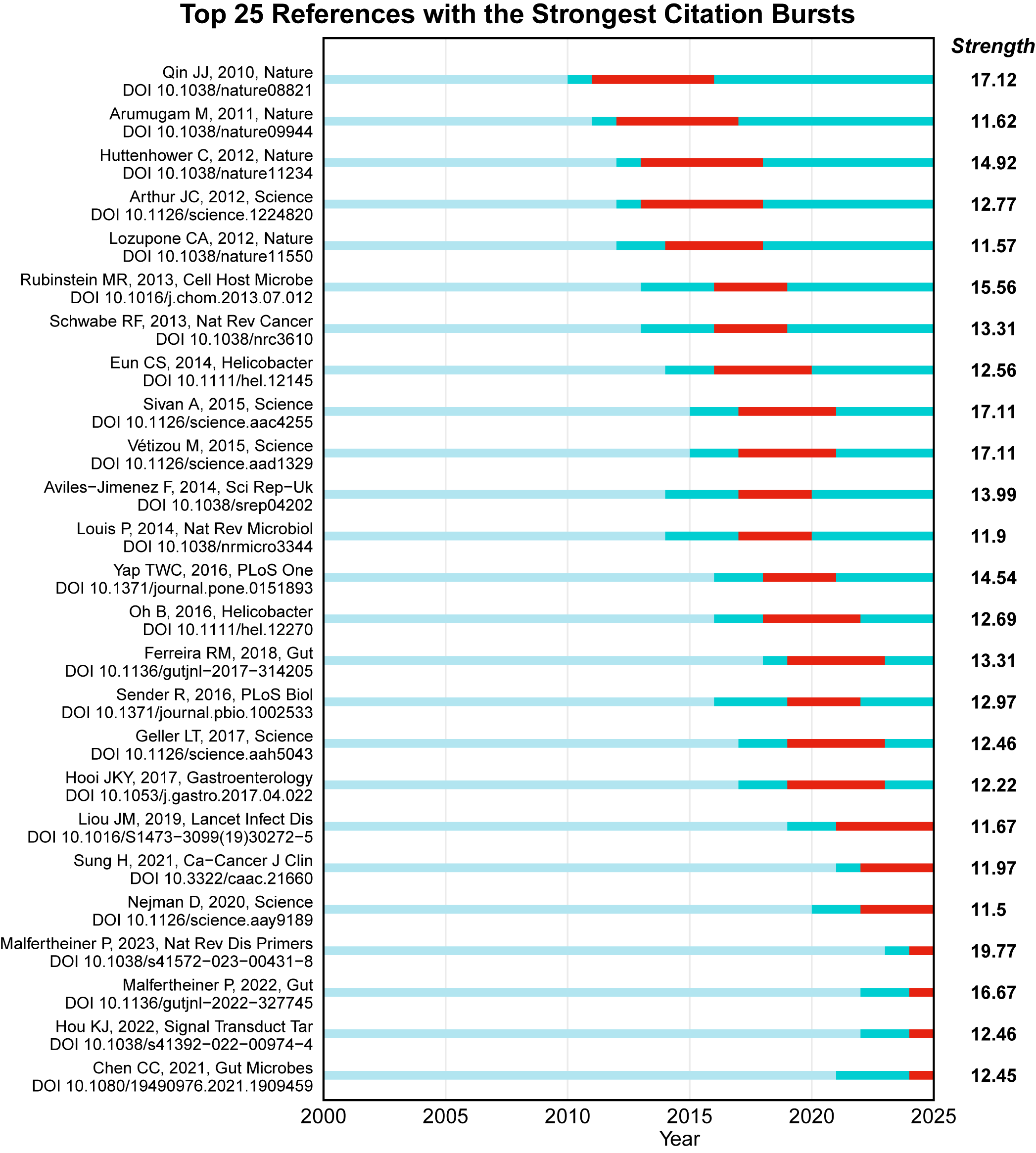

Supplement: Supplementary Figure 10 — Top 25 references with the strongest citation bursts. [file Image10.tif]

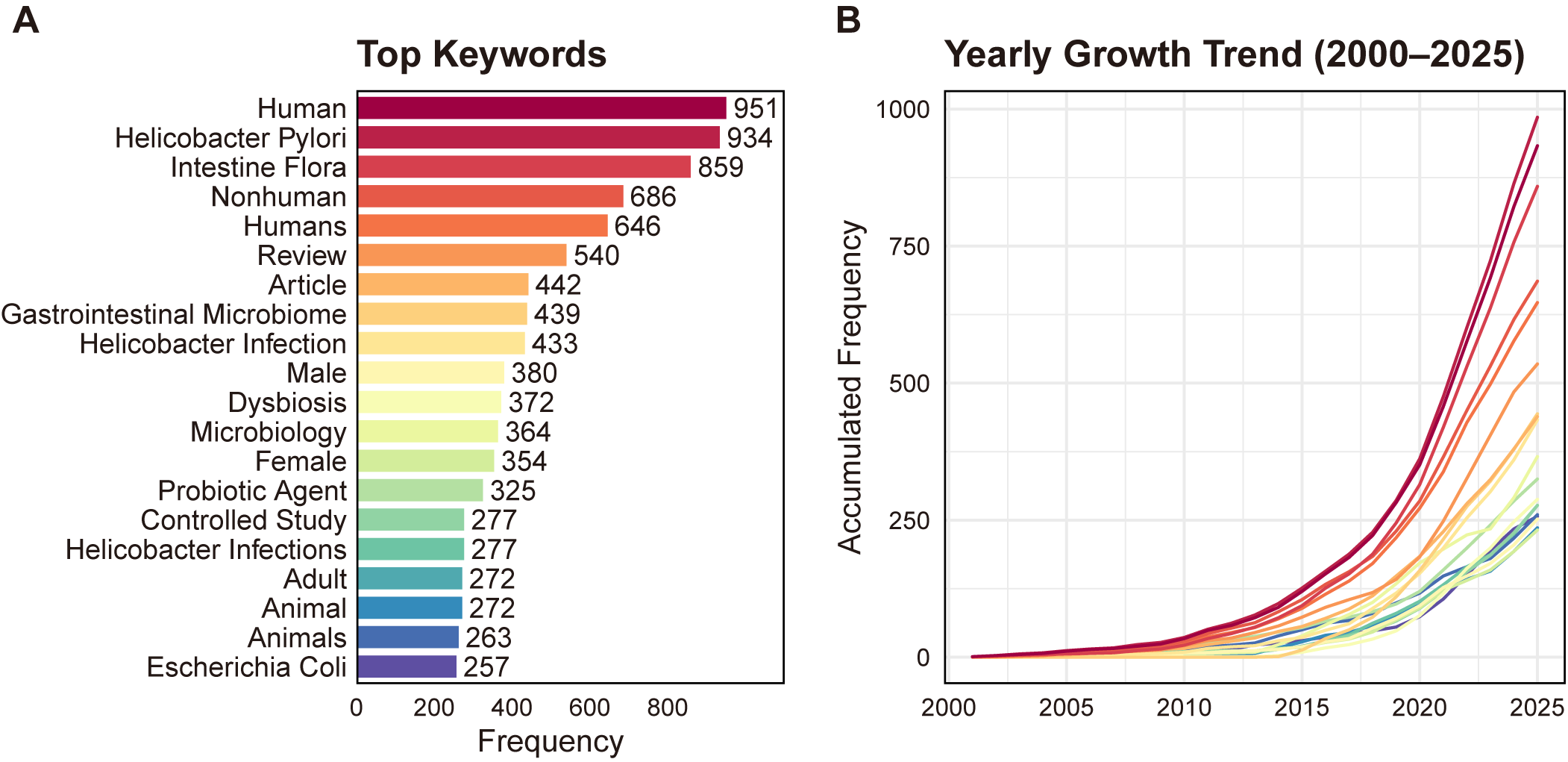

Supplement: Supplementary Figure 11 — Keyword frequency and temporal trends analysis based on Scopus data. (A) Distribution of high-frequency keywords. (B) Accumulated growth trends of top keywords over time. [file Image11.tif]

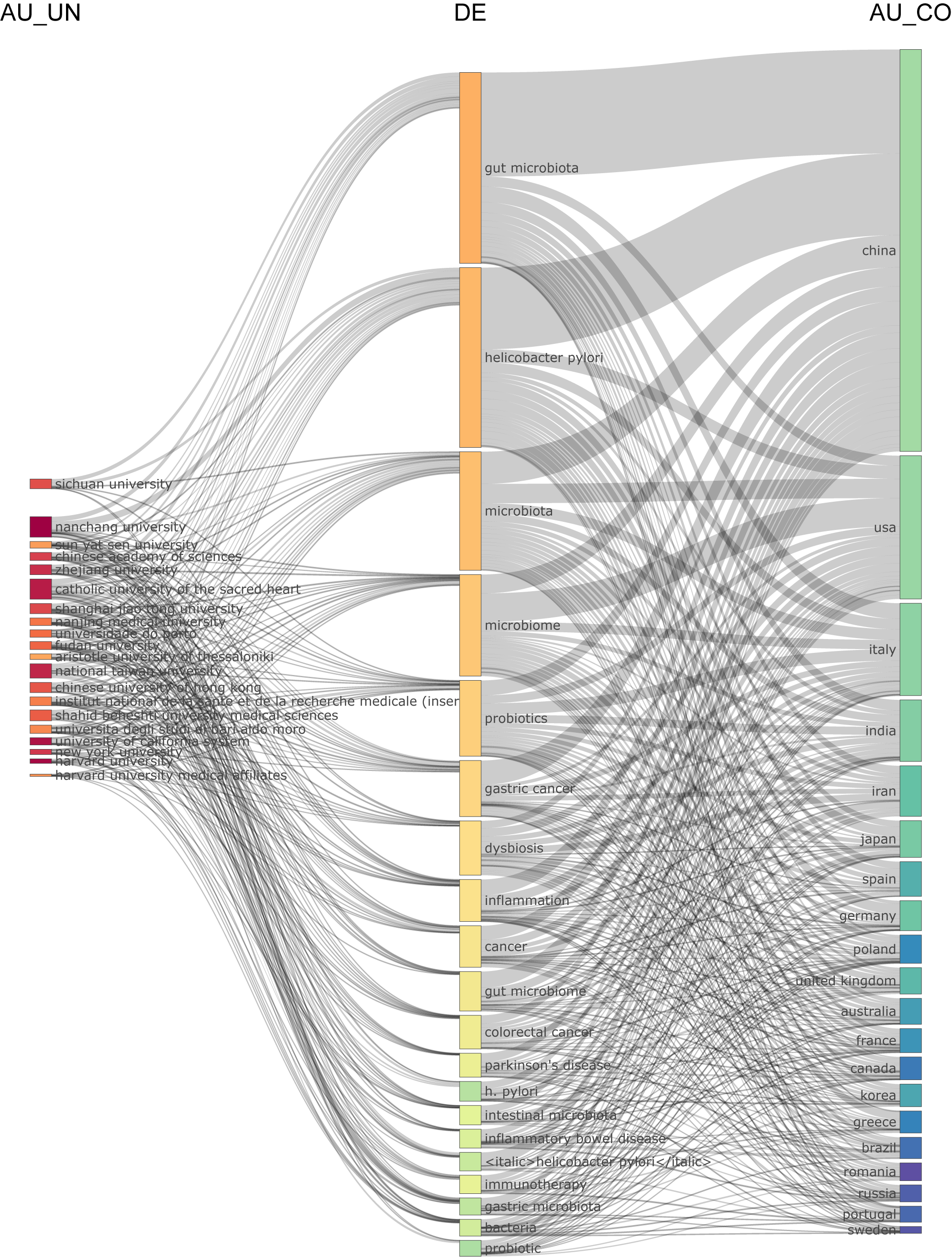

Supplement: Supplementary Figure 12 — Three-field plot of countries, institutions, and keywords. [file Image12.tif]

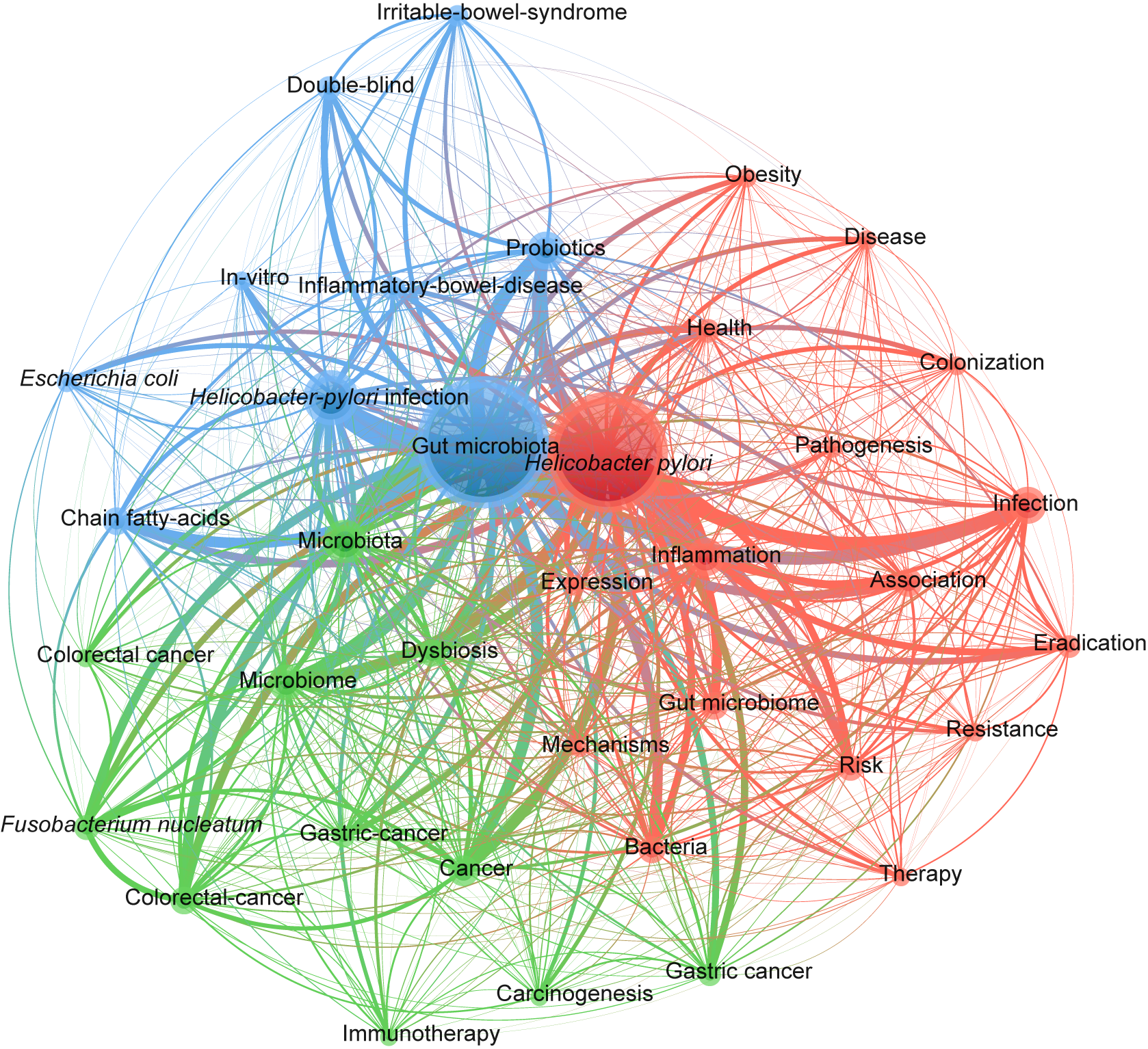

Supplement: Supplementary Figure 13 — Co-occurrence analysis and visualization of keywords. [file Image13.tif]

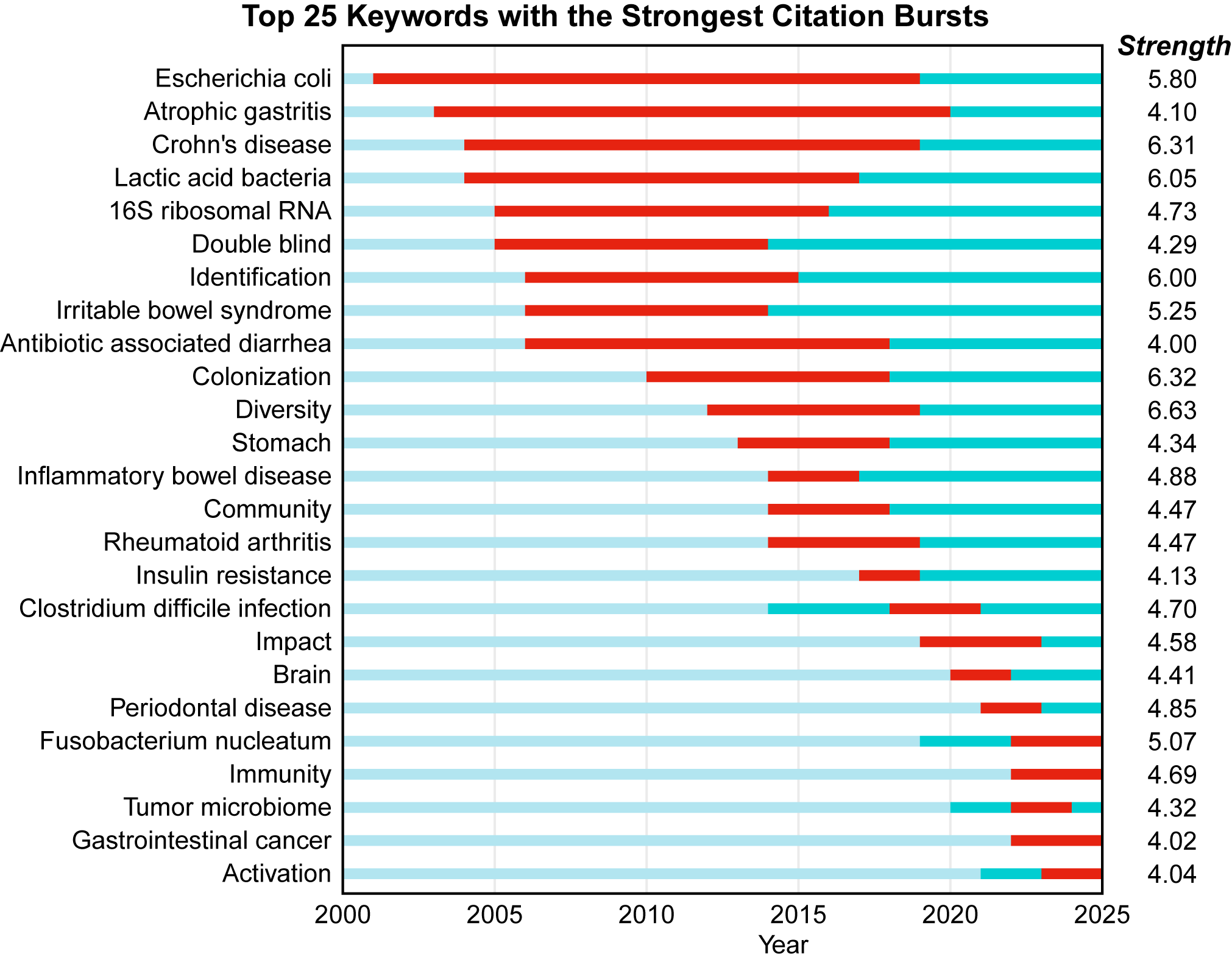

Supplement: Supplementary Figure 14 — Top keywords with the strongest citation bursts. [file Image14.tif]
